# Microbial Communities in Sediments From Four Mildly Acidic Ephemeral Salt Lakes in the Yilgarn Craton (Australia) – Terrestrial Analogs to Ancient Mars

**DOI:** 10.3389/fmicb.2019.00779

**Published:** 2019-05-06

**Authors:** Joost W. Aerts, Rob J. M. van Spanning, Jessica Flahaut, Douwe Molenaar, Phil A. Bland, Matt J. Genge, Pascale Ehrenfreund, Zita Martins

**Affiliations:** ^1^Department of Molecular Cell Biology, Vrije Universiteit Amsterdam, Amsterdam, Netherlands; ^2^Centre de Recherches Pétrographiques et Géochimiques, Centre National de la Recherche Scientifique/Université de Lorraine, Vandœuvre-lès-Nancy, France; ^3^Department of Applied Geology, Curtin University, Perth, WA, Australia; ^4^Department of Earth Science and Engineering, Faculty of Engineering, Imperial College London, London, United Kingdom; ^5^Leiden Observatory, Leiden University, Leiden, Netherlands; ^6^Space Policy Institute, Elliott School of International Affairs, George Washington University, Washington, DC, United States; ^7^Centro de Química-Física Molecular-Institute of Nanoscience and Nanotechnology (CQFM-IN), Institute for Bioengineering and Biosciences (iBB), Departamento de Engenharia Química, Instituto Superior Técnico (IST), Universidade de Lisboa, Lisbon, Portugal

**Keywords:** Yilgarn Craton, microbial communities, 16S sequencing, halophiles, Mars, geology

## Abstract

The Yilgarn Craton in Australia has a large number of naturally occurring shallow ephemeral lakes underlain by a dendritic system of paleodrainage channels. Processes like evaporation, flooding, erosion, as well as inflow of saline, often acidic and ion-rich groundwater contribute to the (dynamic) nature of the lakes and the composition of the sediments. The region has previously been described as an analog environment for early Mars due to its geological and geophysical similarities. Here, we investigated sediment samples of four lake environments aimed at getting a fundamental understanding of the native microbial communities and the mineralogical and (bio)chemical composition of the sediments they are associated with. The dominant mineral phases in the sediments were quartz, feldspars and amphiboles, while halite and gypsum were the only evaporites detected. Element analysis revealed a rich and complex image, in which silicon, iron, and aluminum were the dominant ions, but relative high concentrations of trace elements such as strontium, chromium, zirconium, and barium were also found. The concentrations of organic carbon, nitrogen, and phosphorus were generally low. 16S amplicon sequencing on the Illumina platform showed the presence of diverse microbial communities in all four lake environments. We found that most of the communities were dominated by extremely halophilic Archaea of the *Halobacteriaceae* family. The dynamic nature of these lakes appears to influence the biological, biochemical, and geological components of the ecosystem to a large effect. Inter- and intra-lake variations in the distributions of microbial communities were significant, and could only to a minor degree be explained by underlying environmental conditions. The communities are likely significantly influenced by small scale local effects caused by variations in geological settings and dynamic interactions caused by aeolian transport and flooding and evaporation events.

## Introduction

Understanding the relation between biological and (bio)chemical components in an ecosystem is of major importance in our quest to unravel the origin of life. This holds true when we look at life on Earth, but also when searching for life that may be or may have been present on other planetary bodies in our solar system, most notably on Mars. Life depends, in part, on the availability of water and of organic and/or inorganic compounds essential for free energy transduction and biomass formation. Challenges for life are brought about by extremities in pH, salinity, temperature and pressure, by desiccated conditions, high radiation levels or by complete absence of sunlight. Deserts, caves, salt lakes, acidic hot springs and ‘black smokers’ are just a few examples of such environments found on Earth. Even though these environments seem very hostile to life, we often find that microbial life is plentiful and has been fully adapted to such conditions (Aerts et al., 2014; Martins et al., 2017; Sarbu et al., 2018). These observations suggest that it may not be completely implausible to propose that life was once present on Mars or even that it could still be present in protected strongholds today.

Current conditions on Mars are characterized by extreme aridity, freezing temperatures (average -60°C), oxidizing surface conditions and high radiation levels due to a thin atmosphere, all of which push the limits for potential presence of life (Pavlov et al., 2002; Quinn et al., 2013; Hassler et al., 2014). Nonetheless, there are strong indications that Mars had a more favorable climate to support life during the first billion years after its formation, such as warmer temperatures and the presence of liquid water (Pollack et al., 1987; Squyres et al., 2004; Poulet et al., 2005; Bibring et al., 2006; Mustard et al., 2008; Ehlmann et al., 2011; Grotzinger et al., 2014). Evidence for a global climate change on Mars include the presence of large ancient channels and valleys and water-lain sedimentary rocks, formed in shallow seas, lakes and salt flats (Carr and Head, 2010). Of great importance is also a recent study showing the presence of organic molecules in the Mars Gale crater, suggesting that organic molecules indicative of life (if present on Mars) may have been preserved in certain environments on the red planet (Eigenbrode et al., 2018). Furthermore, simulated Mars conditions show that clay and sulfate minerals preserve amino acids (dos Santos et al., 2016).

The planes of the Yilgarn craton in southern western Australia have been described as terrestrial analog environments for ancient Mars based on mineralogical, geological, and geochemical features (Benison and Bowen, 2006). Hundreds of individual ephemeral saline lakes exist in this region. The waters in these lakes vary strongly in terms of pH and include extremely acidic (pHs < 4), moderately acidic (pH 4–6), neutral (pH 6–8), or moderately alkaline (pH > 8) lake waters and ground-waters. Even where lake waters are neutral or alkaline, ground-waters tend to be extremely acidic (Mann, 1983; McArthur et al., 1989; Lyons et al., 1990; Benison and Bowen, 2006). The highly weathered Archean bedrock in the region is composed of a combination of greenstone belts of igneous and metamorphic rocks, including granite, quartzite, gneiss, anorthite, and ironstone. The ground-waters as well as the shallow lake waters contain high amounts of dissolved solids, such as silicon, magnesium, potassium, sodium, chloride, sulfate, aluminum, bromide, iron, and other (Gray, 2001; Bowen and Benison, 2009). At average, evaporation outweighs (180–280 cm/y) rainfall (26–34 cm/y) in this region, which results in dry conditions in many of the lakes during the largest part of the year. During dry periods, gypsum, halite, kaolinite and iron oxides are precipitated from the lake waters, while flooding events can partly or completely re-dissolve these precipitates again (Benison et al., 2007). This dynamic environment shows similarities to a phase in which Mars is proposed to have undergone a shift from a relatively water-rich era with near-neutral pH, toward a more arid era that became dominated by saline and acidic waters induced by extensive weathering of the basaltic bedrock (Bibring et al., 2006; Chevrier et al., 2007). It could therefore compare to some of Mars sulfates and chloride deposits in term of geologic processes (Osterloo et al., 2008, 2010; Flahaut et al., 2015).

A variety of papers has been attributed to describing the geological, geochemical and mineralogical characteristics of lake environments in the Yilgarn Craton region (Anand and Paine, 2002; Benison and Bowen, 2006; Benison et al., 2007; Bowen and Benison, 2009; Story et al., 2010; Benison, 2013; Bowen et al., 2013; Ruecker et al., 2016). Only a few papers have focused on the microbiology of the region (Benison et al., 2008; Mormile et al., 2009; Johnson et al., 2015). Previous microbial studies in the region have mainly focused on the lake waters (Mormile et al., 2009), although a more recent paper described for the first time the metagenome of sediment samples from a highly acidic saline salt lake in the Yilgarn craton (Johnson et al., 2015). Both studies showed that *Proteobacteria* were the dominant phylum, while *Actinobacteria* and *Bacteroidetes* also contributed significantly to the microbial communities. However, the reason why certain bacterial communities flourish in these relative hostile environments remains not well understood.

In this study, we aim at obtaining deeper insights in the prokaryotic microbial communities present in these types of environments. We also report on the chemical and physical characteristics of those regions. For that, we investigated sediment samples from the non-flooded parts of four geographically separated mildly acidic, saline lake environments in the Yilgarn Craton (Australia). Prokaryotic profiles were determined using Illumina 16S amplicon sequencing. Chemistry and physics integrated X-ray diffraction, organic carbon and nitrogen measurements, and (trace) elements analysis.

## Materials and Methods

### Samples

**FIGURE 1:**
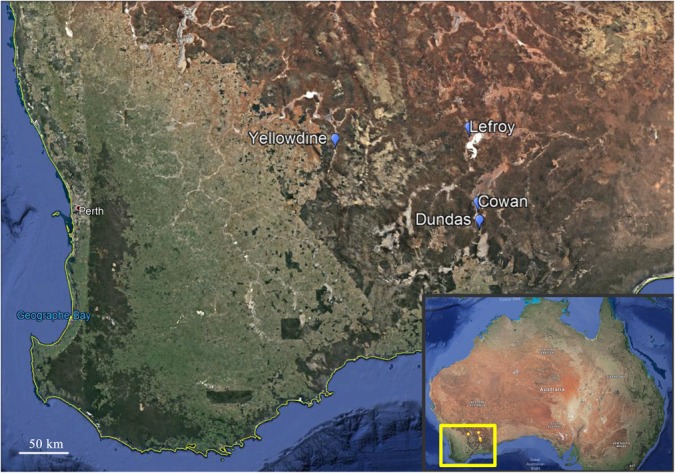
Overview of the locations of the four lakes environments in the southern western part of Australia that were investigated during this study. Locations of Lake Cowan, Lake Dundas, Lake Lefroy, and Lake Yellowdine are indicated on the map with markers. Credits: Google Earth.

**FIGURE 2:**
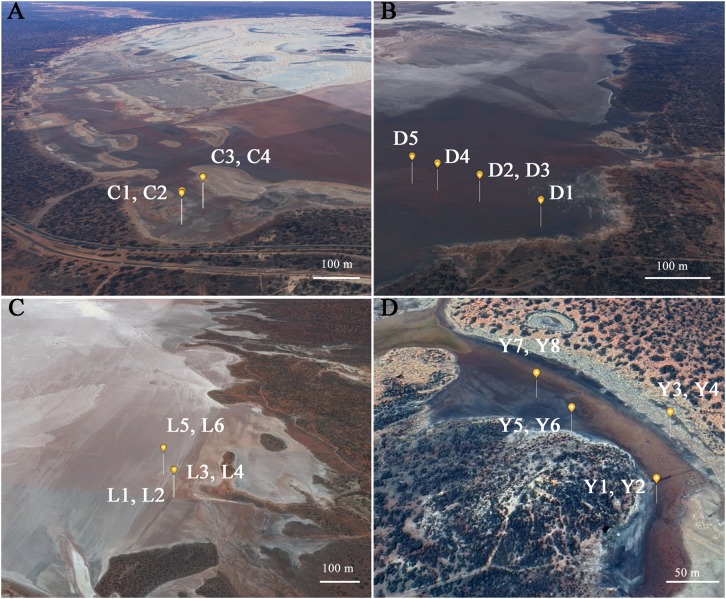
Detailed view from the specific sampling location per lake environment. **(A)** C, Lake Cowan; **(B)** D, Lake Dundas; **(C)** L, Lake Lefroy; **(D)** Y, Lake Yellowdine. Credits: Google Earth.

A total of 23 sediment samples were collected (August 2013) from the non-flooded parts of four ephemeral lakes in the Yilgarn Craton, namely: Lake Lefroy (L samples), Lake Cowan (C samples), Lake Dundas (D samples), and Lake Yellowdine (Y samples) (Figure 1, 2). Except for Lake Yellowdine (which had no official name but was located in the Yellowdine nature reserve), all lakes are referred to by their official name. We selected multiple sample sites in each lake area in order to determine whether there is variation within the same lake site. We collected surface and subsurface samples for most sample sites in order to compare potential differences related to depth. For subsurface samples, we used a shovel to dig to 30 cm depth, where after the sample was collected. If digging was not possible due to a hard subsurface we only collected a surface sample. For more information on the sample characteristics and location, see Table 1. All samples were collected using sterilized spatulas (sterilization of the material was achieved by wrapping up in aluminum foil and heating at 500°C for at least 3 h). After collecting, samples were stored in sterile 50 mL sterile falcons from Sterilin (United Kingdom). Soil pH was measured on site using a Gardman soil pH meter. After shipment, samples were stored at -20°C until further processing.

**Table 1:** Sample site descriptions for sediments from Lake Cowan (C), Lake Dundas (D), Lake Lefroy (L), and Lake Yellowdine (Y).

Sample	Site	Depth (cm)	Soil pH	GPS coordinates	Elevation
C1	Lake Cowan	0	6.0	S32.16084, E121.76917	269 m
C2	Lake Cowan	30	6.0	S32.16084, E121.76917	269 m
C3	Lake Cowan	0	6.0	S32.16167, E121.76797	264 m
C4	Lake Cowan	30	6.2	S32.16167, E121.76797	264 m
D1	Lake Dundas	0	6.0	S32.38191, E121.79113	248 m
D2	Lake Dundas	0	6.0	S32.38282, E121.79294	248 m
D3	Lake Dundas	30	6.0	S32.38282, E121.79294	248 m
D4	Lake Dundas	0	6.9	S32.38319, E121.79433	250 m
D5	Lake Dundas	0	7.3	S32.38343, E121.79523	248 m
L1	Lake Lefroy	0	5.0	S31.23428, E121.64400	294 m
L2	Lake Lefroy	30	6.0	S31.23428, E121.64400	294 m
L3	Lake Lefroy	0	6.0	S31.23429, E121.64406	293 m
L4	Lake Lefroy	30	6.0	S31.23429, E121.64406	293 m
L5	Lake Lefroy	0	6.0	S31.23615, E121.64480	295 m
L6	Lake Lefroy	30	6.0	S31.23615, E121.64480	295 m
Y1	Lake Yellowdine	0	6.0	S31.36019, E119.70271	366 m
Y2	Lake Yellowdine	30	6.0	S31.36019, E119.70271	366 m
Y3	Lake Yellowdine	0	4.5	S31.35980, E119.70480	359 m
Y4	Lake Yellowdine	30	6.0	S31.35980, E119.70480	359 m
Y5	Lake Yellowdine	0	6.0	S31.35787, E119.70403	359 m
Y6	Lake Yellowdine	30	6.0	S31.35787, E119.70403	359 m
Y7	Lake Yellowdine	0	6.1	S31.35659, E119.70491	357 m
Y8	Lake Yellowdine	30	6.9	S31.35659, E119.70491	357 m

### DNA Extraction

DNA was extracted from 0.5 g of each homogenized sediment sample using the MOBIO PowerSoil extraction kit (MOBIO Laboratories Inc., Carlsbad, CA, United States), with adaptations to the extraction protocol according to Direito et al. (2012). These adaptations were included to minimize adsorption of extracted DNA by the mineral matrix. Extraction blanks were incorporated during the procedure to assess possible contamination during the extraction process. All handling of the samples and negative controls during the extraction process were conducted in a UV3 HEPA PCR workstation (UVP, Upland, CA, United States), equipped with a HEPA filter and a UV illuminator to prevent extraneous DNA contamination. Concentration of the different extracts were determined using a Quant-iT high-sensitivity DNA assay kit and a Qubit^^®^^ fluorometer (Invitrogen, Carlsbad, CA, United States). DNA extracts were stored at -20°C until further processing.

### Illumina 16S Amplicon Sequencing

All DNA extracts were diluted to a concentration of 0.1 ng/μl prior to PCR amplification. If the concentration of the DNA extract was lower than 0.1 ng/μl it was processed undiluted. PCR reactions were performed in triplicate using Phusion Green Hot Start II High-Fidelity DNA Polymerase (Thermo Fisher Scientific, Sweden). We targeted the V3-V4 region of the 16S rRNA gene, using the V3 forward primer S-D-Bact-0341-b-S-17, 5′-CCTACGGGNGGCWGCAG-3′ (Herlemann et al., 2011) and the V4 reverse primer S-D-Bact-0785- a-A-21, 5′-GACTACHVGGGTATCTAATCC-3′ (Muyzer et al., 1993), giving rise to ∼430 bp long dsDNA fragments. The primers were dual barcoded and were compatible with Illumina sequencing platforms as described previously (Caporaso et al., 2011). Performance of the PCR reaction was checked by running incorporated positive and negative controls from each triplicate plate on 1.5% (w/v) agarose gels. Triplicate PCR products were combined, and each of these was purified using SPRI beads (Agencourt^^®^^ AMPure^^®^^ XP, Beckman Coulter, CA, United States). The DNA concentration in the purified samples was determined as described above. Samples were diluted to identical concentrations of 2 ng/μl prior to pooling the diluted PCR products together in equal volumes (10 μl) in one composite sample (including positive and negative controls).

The composite samples were paired-end sequenced at the Vrije Universiteit Amsterdam Medical Center (Amsterdam, Netherlands) on a MiSeq Desktop Sequencer with a 600-cycle MiSeq Reagent Kit v3 (Illumina) according to manufacturer’s instructions. High-throughput sequencing raw data were demultiplexed using bcl2fastq software version 1.8.4 (Illumina) and primers were trimmed using Cutadapt (Martin, 2011). Demultiplexed samples were further processed using a modified version of the Brazilian Microbiome Project 16S profiling analysis pipeline (Pylro et al., 2014). Paired-end reads were joined using PANDAseq (Masella et al., 2012) allowing for a minimum overlap of 30 nucleotides between the forward and reverse reads, a minimum sequence length of 285 and no mismatches in the primer region were allowed. PANDASeq addresses mismatches in overlapping regions by selecting the nucleotide with the best sequencer-assigned quality score. Because PANDAseq incorporates a base quality filter during read assembling, the threshold for consecutive high-quality bases per read was set to zero. Metadata and demultiplexed samples were merged using add_qiime_labels.py (Caporaso et al., 2010) and sequence headers were changed using bmp-Qiime2Uparse.pl (Pylro et al., 2014). UPARSE was used to dereplicate, filter chimeras, discard OTUs (operational taxonomic units) detected less than two times and OTU clustering at 97% similarity (Edgar, 2010, 2013). The OTU taxonomy was assigned using the UCLUST algorithm (Edgar, 2010) on QIIME (Caporaso et al., 2010) using SILVA compatible taxonomy mapping files (Silva database release 128) (Quast et al., 2013; Yilmaz et al., 2014) and aligned using align_seqs.py in QIIME (Caporaso et al., 2009). Taxonomy was manually curated and refined up to genus level based on 97% similarity of reference sequences. The reference tree was calculated using the make_phylogeny script in QIIME (Price et al., 2009). We generated a BIOM file using make_otu_table.py on QIIME (Caporaso et al., 2010). Prior to further analysis we produced an OTU table and a taxonomy table using BIOM scripts (McDonald et al., 2012). The OTUs detected in negative controls and procedural blanks were manually removed from the dataset.

After quality filtering, taxonomy assignment (97% identity), subtraction of contaminating sequences and removal of singletons, the dataset comprised 1,817,287 reads which were attributed to a total of 4,270 OTUs. Next, a filtering step was performed as described by Bokulich et al. (2013), where OTUs representing fewer than 0.005% of the total reads were removed from the dataset. This trimmed dataset resulted in 1,748,123 reads and a total of 1,315 OTUs (Supplementary Table S1).

### Mineralogical Analysis

Semi-quantitative X-ray diffraction (XRD) analyses were conducted to determine the composition and relative abundance of the crystalline phases in the bulk sediments. The samples were dried at 70°C for 48 h, ground in a bead-mill to a particle size <100 μm and measured using a Bruker D8 Advance diffractometer equipped with an X-ray Cu source at the Centre de Diffractométrie of the University Lyon, France. Disoriented measurements were made over a 2Theta range of 3°–70°. Determining the presence and identification of clays could not be done accurately with the used approach, so these will be largely left out of the analysis. XRD patterns were analyzed using the Bruker DIFFRAC.SUITE EVA software. Mineralogical fits were performed by comparing D-spacing values to those of minerals listed in the International Center for Diffraction Data database and the Crystallography Open Database (Kabekkodu et al., 2002; Gražulis et al., 2009): mineral abundance was determined as weight percent (wt. %) with a 10 to 20% accuracy.

### Chemical Analysis

Major and trace element analysis were performed at the Service d’Analyse des Roches et des Mineraux (CNRS – CRPG) in Nancy, France. Trace elements were analyzed using ICP-MS and major elements with ICP-OES. FeO was measured by potentiometric titration with K_2_Cr_2_O_7_ (potassium dichromate).

### Carbon and Nitrogen Measurements

Samples were powdered using heat sterilized mortar and pestle and subsequently dried at 60°C for 24 h. Analysis was carried out using a FlashEA 1112 (Thermo Scientific), with EAGER300 operating software and a thermo conductive detector. For carbon and nitrogen measurements, approximately 30 mg of each sample was weighed in silver holder cups. An empty holder cup was used as a blank. Two to three milligram of EDTA was used as standard (7.522% N, 32.237% C, 4.836% H, 34.386% O). For the removal of inorganic carbon, cups containing the samples were placed upright in a desiccator containing 12M HCl. Samples were exposed overnight to the vapors. Complete removal of inorganic carbon was tested by adding 10 μl of 10% HCl to a cup in order to determine if vapors were produced (which would indicate presence of more inorganic carbon). This procedure was repeated until no further fumes were produced. Samples were dried at 80°C prior to CN measurements. The silver cups were sealed off and placed within a tin cup, which was sealed prior to measurement. Amounts are measured as percentage per weight and measurements were carried out in duplicate.

## Results

### Overview of the Microbial Communities in the Lake Sediments

**FIGURE 3:**
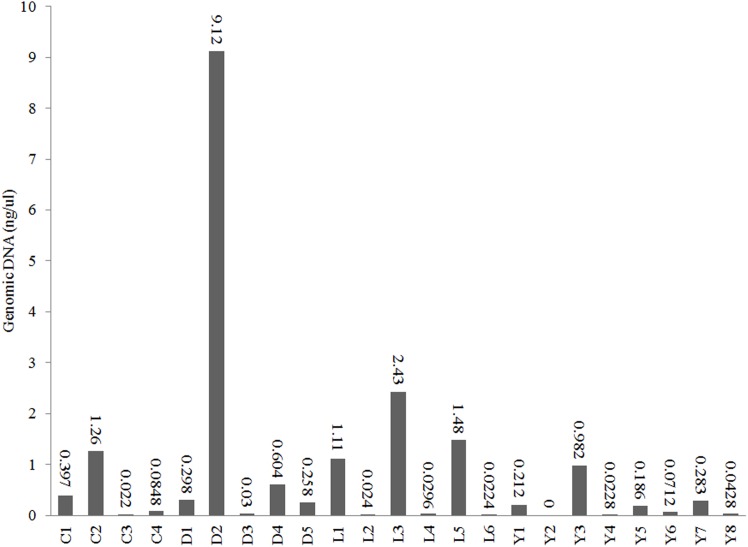
DNA concentrations in the extracts of the sediments from lakes Cowan (C), Dundas (D), Lefroy (L), and Yellowdine (Y).

We used Illumina 16S amplicon sequencing to obtain a detailed insight in the prokaryotic community structures of the 23 sediment samples (Table 1). All sediment samples contained detectable amounts of extractable DNA except for one subsurface sample taken from Lake Yellowdine (Figure 3). Generally, surface samples contained considerably more DNA (one to two factors of magnitude) than subsurface samples. Differences in initial DNA are also reflected in the final number of reads obtained for each sample (Supplementary Table S1). Some large differences observed between the number of reads is therefore interpreted as a reflection of naturally occurring differences in biomass, rather than potential artifacts introduced during subsequent experimental steps.

**FIGURE 4:**
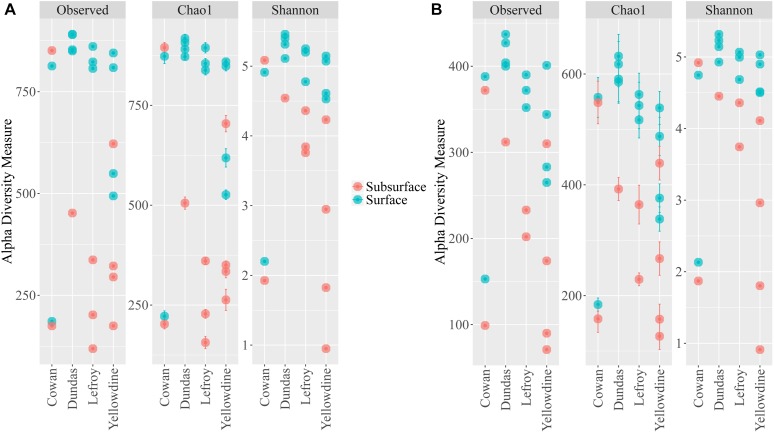
Alpha diversity metrics for the **(A)** filtered non-rarefied 16S dataset and the **(B)** rarefied 16S dataset. Samples are colored according to sampling depth and spatially separated according to sampling location.

**FIGURE 5:**
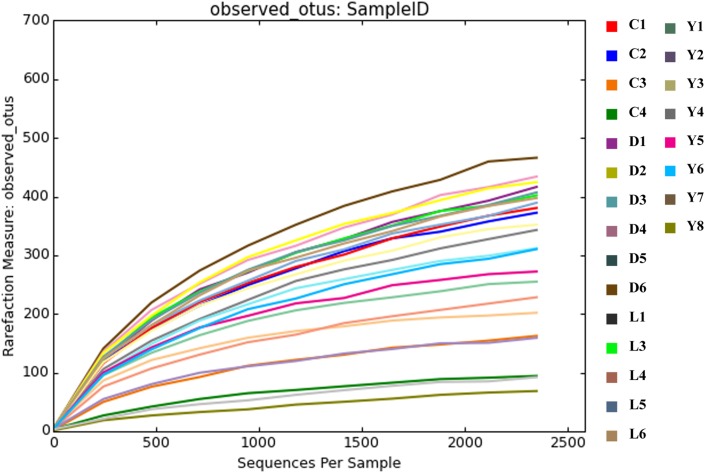
Rarefaction curves depicting the coverage of species after the rarefaction step. The rarefaction plot was produced using QIIME software.

Alpha diversity measurements were performed using the plot_richness function from the Phyloseq package in R (Figure 4A). In order to equalize library sizes for downstream analysis, a rarefaction step with a sampling depth of 2350 was performed (see Figure 5 for the rarefaction curves). One subsurface sample from Lake Lefroy (L2), which had only 820 sequence reads, was excluded and hence 22 of the 23 investigated samples are included in the rarefied dataset. Alpha diversity of the samples decreased after rarefaction (Figure 4B), while only a very small fraction of total OTU’s (operational taxonomic units) was removed from the dataset (Supplementary Table S1). This is explained by the observed loss of table density after rarefaction, indicating that a larger fraction of the OTUs is represented in lower abundances and, as a result, is represented in fewer samples (but still included within the dataset). We found highest alpha diversity in surface samples, which is in line with higher yields of extractable DNA in these samples. It seems that higher biomass correlates with a higher species diversity. Furthermore, this apparent correlation is of a logarithmic nature (i.e., disproportionally more DNA is needed to obtain a few extra species) (Supplementary Figure S1). However, it must also be noted that some plant matter was observed on site which is degraded less efficiently in surface soils. This could also explain higher DNA concentrations in the extracts since at the point of measurement no distinction is made between prokaryotic or eukaryotic DNA. If the higher abundances of DNA would be a result of non-microbial material it may facilitate an extra source of food which could also explain higher species diversity in these samples.

**FIGURE 6:**
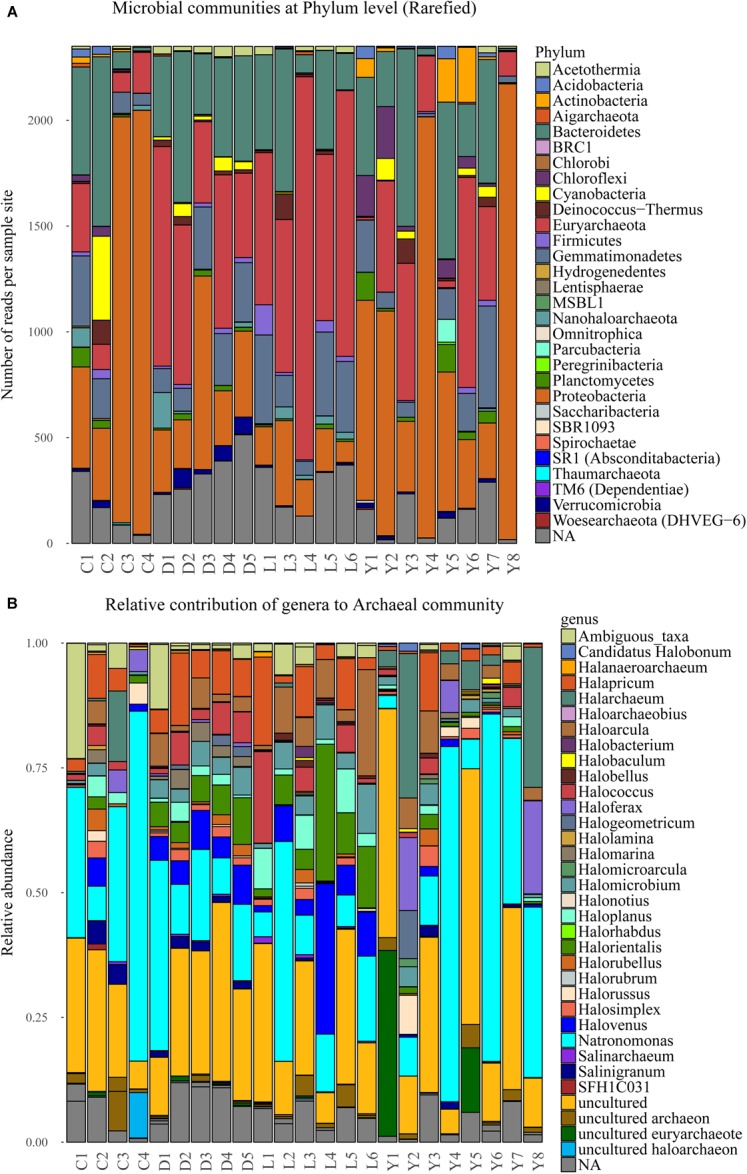
**(A)** Stacked bar depicting the microbial composition at phylum level in the sediment samples of the rarefied dataset. **(B)** Normalized stacked bar plot of the archaeal community within the sediments shown at genus level. C, Lake Cowan; D, Lake Dundas; L, Lake Lefroy; Y, Lake Yellowdine.

The bulk of the sequence reads (76%) originates from four dominant phyla, namely: *Euryarchaeota* (23%), *Proteobacteria* (22%), *Bacteroidetes* (22%), and *Gemmatimonadetes* (9%). Another 11% of the sequences are not related to known phyla while the remainder of reads (13%) is shared between 27 phyla (Figure 6A). The extremely halophilic Archaeal family *Halobacteriaceae* (phylum *Euryarchaeota*) dominates the dataset at family level comprising approximately 23% of the reads and representing over 30 known genera. The archaeal community profile in each sediment sample is specified in Figure 6B. Most of the samples contain similar (in terms of species represented) profiles for the family of *Halobacteriaceae*. However, the relative contribution of *Halobacteriaceae* differs significantly between samples from different lake environments. Lake Dundas and Lake Lefroy sediments have consistently the highest contribution of *Halobacteriaceae* members while Lake Cowan sediments show much lower numbers in general. In Lake Yellowdine there is strong variation in the contribution of *Halobacteriaceae* between samples.

Overall, a large part of the detected bacterial genera have similarity with species of marine origin and may therefore have tolerance (and need) for higher salt concentrations (e.g., 6.8% *PAUC43f Marine-benthic-group bacteria*, 3.3% *Oceanospirillales*, 2.8% *Marinobacter*, 0.5% *Planctomyces)*. More extreme halophilic bacteria are also abundant representing species that belong to the genera of *Salinisphaera* (6.1%) and *Salinibacter* (5.1%) as the most dominant ones. A number of samples from Lake Cowan and Lake Yellowdine contained two species that were relatively abundant (in between 5 and 80% contribution). These were *Salinisphaera* and *Marinobacter*, and they coexist in most of these samples (data not shown). Notably, these two species were hardly present in the Dundas and Lefroy sediments. Observed variations in microbial communities are greatest in lakes Cowan and Lake Yellowdine while lakes Dundas and Lefroy have less in-lake variation and also share more similar profiles with each other.

Most other species are detected in lower abundances. These include species involved in the nitrogen and sulfur cycles (most notably purple sulfur bacteria), but a wide variety of phylotypes is seen. A relative large fraction of the obtained reads could not be assigned to known taxa (where the identity threshold was set at 0.97) (Supplementary Figure S2), indicating that there are still many unknown species in these types of environments One example is an uncultured *Gammaproteobacterium* which makes up almost 50% of the reads of a sample from Lake Yelowdine (Y4).

### Effect of Location and Depth of the Samples on the Composition of the Microbial Communities

**FIGURE 7:**
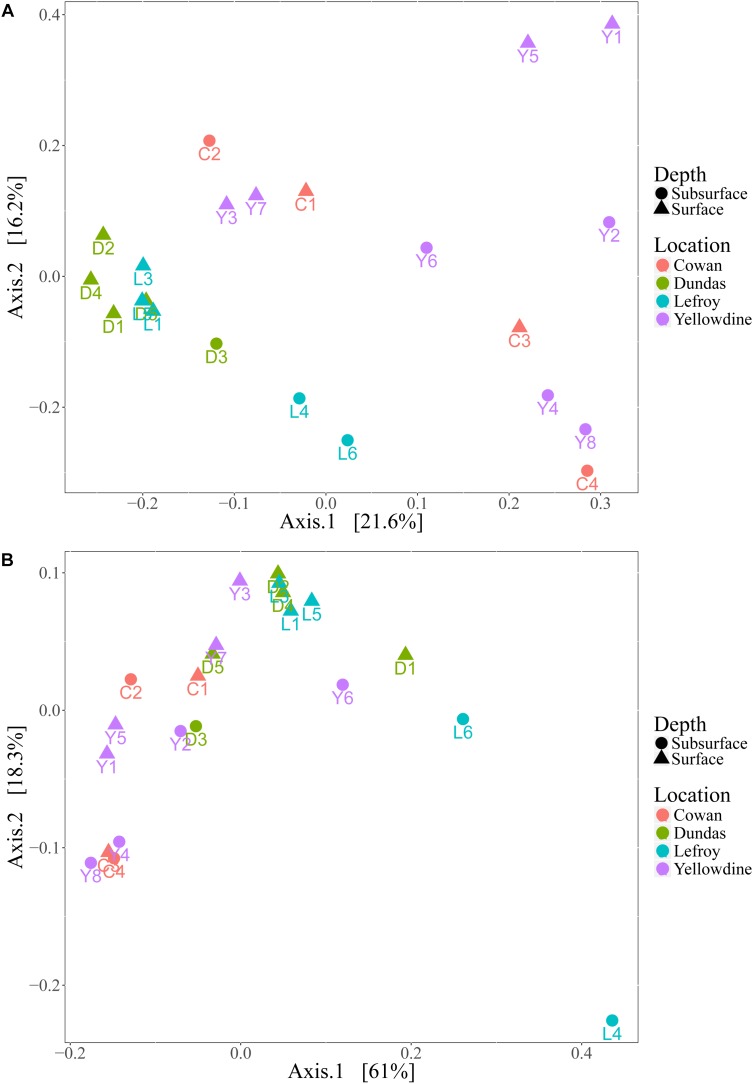
**(A)** Principal coordinate analysis (PCoA) plot using unweighted unifrac metric and **(B)** weighted unifrac metric. Samples are colored with respect to the lake they were sampled from and are shaped according to sampling depth.

Although the investigated lake environments are geographically separated from each other, the prokaryotic communities in the different samples generally share a large number of phylotypes. There are differences in relative contributions of these taxa between the samples, but these are not always indicative for the sampled lake environment. Even more so, microbial variation between samples within the same lake site is often greater than variation between samples of different lake site sediments. Principal coordinates analysis (PCoA) plots were used to visualize this variation by ordinating the data, using weighted and unweighted unifrac metrics (Figure 7). The unweighted variant ordinates samples solely based on the variation in the types of species that are present, while the weighted variant also includes the effect of the relative abundances of those species in the samples.

Samples from Lake Dundas and Lake Lefroy form a more separated and somewhat denser cluster in the unweighted PCoA plot (Figure 7A). This indicates that samples from these two environments have lower intra-lake variation and are also more similar to each other in terms of species that are represented when compared to samples from Lake Cowan and Lake Yellowdine. The spread observed in samples from the other two lake environments is much higher and reveals strong intra- and inter-lake variation. However, using unweighted metrics only explains a small percentage of the variation that is present within the dataset, as is shown by the low variation percentages at the primary and secondary axis.

The weighted PCoA plot (Figure 7B) shows a different distribution in which much more overlap is found between samples from the different lake environments. More of the variation within the microbial communities could be explained by also inferring the quantitative characteristics of the communities. However, rather than pulling the communities apart, it formed denser clusters, making interpretation more complex. One sample from Lake Lefroy (L4) was more separated from the bulk. This is likely because it had the highest contribution of *Halobacteriaceae* from all the samples, with especially high abundances of the genera *Haloorientalis* and *Halovenus* (together making up 50% of all the reads in this sample).

We performed Adonis tests in order to determine whether the sampling location (i.e., lake environment) could explain part of the observed variation in the microbial communities. (ADONIS: Weighted UniFrac *R*^2^ = 0.34, *p* = 0.005; Unweighted UniFrac *R*^2^= 0.26, *p* = 0.001). The *R*^2^ value (effect size) shows the percentage of variation explained by the implemented variable (lake environment), while the p-value determines the statistical significance of the test. Although the *p*-values for both Adonis tests are significant and the effect sizes were reasonably high, strong dispersion within the sample groups was also observed. A test for homogeneity of dispersion showed a significant *p*-value (0.009), indicating that the statistically significant effect found by the Adonis tests, may also be a result of heterogeneous dispersion within sample groups.

We also conducted Adonis tests to determine the effect of sampling depth on the microbial community structures of the samples (Unweighted UniFrac *R*^2^ = 0.10, *p* = 0.011; Weighted Unifrac *R*^2^ = 0.087, *p* = 0.115). This revealed that the *p*-value for the unweighted unifrac metric was significant, but only with a small effect size, explaining about 10% of the variation in distances in the unweighted unifrac ordination. Permutation tests for homogeneity of dispersion resulted in insignificant *p*-values (Unweighted Unifrac *p* = 0.53, Weighted Unifrac *p* = 0.208). These data indicate that the depth of the sample has a small but statistically significant effect on the type of species that are present, but no significant effect on their relative abundances.

### Organic Carbon and Nitrogen

Overall, organic carbon and nitrogen abundances in the sediments were low (Table 2), and higher carbon or nitrogen in the samples did not necessarily result in higher amounts of DNA in the samples. Adonis tests came up with significant *p*-values for carbon and nitrogen using the unweighted unifrac metric (Carbon: *R*^2^ = 0.079, *p* = 0.034; Nitrogen: *R*^2^ = 0.082, *p* = 0.033) while weighted unifrac analysis resulted in insignificant *p*-values. However, the effect sizes (*R*^2^ < 0.1) of the Adonis tests were very small, suggesting that carbon and nitrogen load in the sediments only explains a small portion of the variation observed between the different communities of the sediments. C/N ratios varied strongly between the samples with values ranging from 8 to 22 regardless of the type of lake they were taken from (Table 2).

**Table 2:** Organic carbon and nitrogen contents (weight %) and C/N ratios of the sediments samples from Lake Cowan (C), Lake Dundas (D), Lake Lefroy (L), and Lake Yellowdine (Y).

	Carbon	Nitrogen	C/N
C1	0.109 (0.005)	0.0061 (0.0003)	18.33
C2	0.294 (0.005)	0.0259 (0.0003)	11.15
C3	0.1075 (0.005)	0.0048 (0.0003)	22.00
C4	0.081(0.002)	0.0045 (0.0009)	20.00
D1	0.1005 (0.001)	0.0075 (0.0002)	14.29
D2	0.3325 (0.001)	0.0313 (0.0016)	10.65
D3	0.09 (0.008)	0.0079 (0.0019)	11.25
D4	0.0985 (0.003)	0.0069 (0.0008)	14.29
D5	0.0765 (0.002)	0.0048 (0.0008)	16.00
L1	0.1945(0.002)	0.0156 (0.0013)	11.88
L2	0.0805 (0.001)	0.0057 (0.0009)	13.33
L3	0.189 (0.001)	0.0187 (0.0005)	10.00
L4	0.0925 (0.001)	0.0054 (0.0001)	18.00
L5	0.243 (0.002)	0.0220 (0.0005)	10.91
L6	0.092 (0.002)	0.0058 (0)	15.00
Y1	0.3905 (0.001)	0.0330 (0.0005)	11.82
Y2	0.155 (0.001)	0.0148 (0.0009)	10.67
Y3	0.0665 (0.002)	0.0057 (0.0006)	11.67
Y4	0.0725 (0.004)	0.0087 (0.0010)	7.78
Y5	0.2415 (0.001)	0.0212 (0.0007)	11.43
Y6	0.042 (0.005)	0.0033 (0.0016)	13.33
Y7	0.183 (0.004)	0.0142 (0.0008)	12.86
Y8	0.1735 (0.004)	0.0135 (0.0013)	13.08

### X-Ray Diffraction Analysis

**FIGURE 8:**
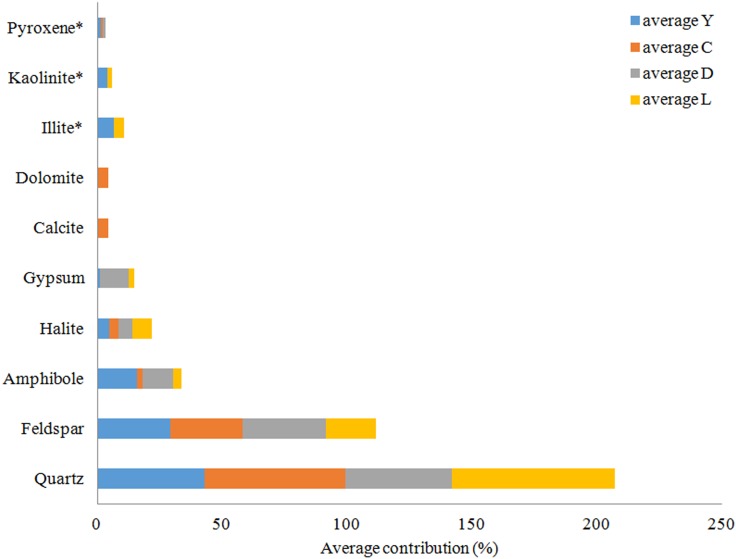
Average mineral distribution per lake in percentages. Y, Lake Yellowdine; C, Lake Cowan; D, Lake Dundas; L, Lake Lefroy. ^∗^Clay minerals, no accurate quantification possible.

An overview of the key compositions of the analyzed sediment samples is given in Table 3 and Figure 8. Overall, the samples have a high crystallinity index, making the XRD procedure more accurate for the whole rock analysis. Quartz is by far the most dominant mineral phase (contributing 51% on average) with the exception of several samples from Lake Yellowdine in which feldspars and amphiboles dominate. On average, feldspars contribute 28% and amphiboles about 10% to the total of the sediment samples. Halite (NaCl), being the most dominant evaporate mineral, contributes about 5.5% on average and its prominent detection reflects the saline nature of the waters that overlay these sediments during flooding events. These mineral phases are observed in all samples, albeit in different proportions. Gypsum (CaSO_4_) is detected in samples from Lake Dundas and Lake Lefroy as well as in one sample of Lake Yellowdine, although only in large quantities (making up around 15%) in two Lake Dundas samples (D4 and D5). The carbonates dolomite (CaMg(CO_3_)_2_) and calcite (CaCO_3_) are observed exclusively in Lake Cowan.

**Table 3:** Semi-quantitative mineralogical profiles as determined by XRD for the sediment samples of Lake Cowan (C), Lake Dundas (D), Lake Lefroy (L), and Lake Yellowdine (Y).

	Crystalline	Amorphous	Quartz	Feldspar^a^	Halite	Amphibole^b^	Calcite	Dolomite	Gypsum	Kaolinite^∗^	Illite^∗^	Pyroxene^∗^	Silicates
C1	89	11	64	21	2	2	6	5	0	0	0	0	0
C2	84	16	48	39	3	1	3	6	0	0	0	0	0
C3	85	15	63	22	3	3	4	4	0	0	0	1	0
C4	85	15	50	33	6	3	4	3	0	0	0	0	1
D1	77	23	38	31	7	24	0	0	0	0	0	0	0
D2	85	15	50	33	6	8	0	0	3	0	0	0	0
D3	73	27	46	34	8	11	0	0	0	0	0	1	0
D4	78	22	39	28	5	10	0	0	18	0	0	0	0
D5	78	22	37	36	4	9	0	0	14	0	0	0	0
L1	80	20	58	24	6	4	0	0	2	2	4	0	0
L2	76	24	66	20	8	4	0	0	0	2	0	0	0
L3	84	16	79	9	6	2	0	0	2	2	0	0	0
L4	77	23	70	19	7	3	0	0	0	1	0	0	0
L5	68	32	58	23	9	5	0	0	3	2	0	0	0
L6	75	25	62	24	9	3	0	0	0	2	0	0	0
Y1	58	42	45	27	2	8	0	0	0	8	9	1	0
Y2	54	46	19	42	10	9	0	0	0	9	9	2	0
Y3	86	14	59	31	2	6	0	0	1	1	0	0	0
Y4	59	41	54	20	9	12	0	0	0	5	0	0	0
Y5	80	20	45	34	1	18	0	0	0	1	0	1	0
Y6	90	10	84	10	1	3	0	0	0	0	2	0	0
Y7	85	15	23	26	3	47	0	0	0	1	0	0	0
Y8	56	44	15	45	11	25	0	0	0	4	0	0	0

*^a^Best fit for feldspar (anorthite). ^b^Best fit for amphibole (actinolite). ^∗^Clay minerals should be viewed only qualitatively due to inaccurate estimations for quantification.*

We found the most amorphous samples in Lake Yellowdine sediments. This lake is the most clay-rich which is confirmed by the more prominent detection of kaolinite and illite in several samples. Although the technique we used does not allow for quantification of clay minerals (due to their preferred orientation), the presence or absence of these minerals can still be assumed from specific XRD peaks. Kaolinite and illite are also present in some sediments of Lake Lefroy but not in Lake Dundas or Lake Cowan. Trace levels of pyroxenes (aegirine) are seen in some of the sediment samples (<1%). Anorthite (CaAl_2_Si_2_O_8_) was the best fit for the (plagioclase) feldspar which is the calcium-rich end member of the group. Actinolite (Ca_2_(Mg_4.5-2.5_Fe^2+^_0.5-2.5_)Si_8_O_22_(OH)_2_) best matches the amphibole peaks and is an intermediate member in a solid-solution series between magnesium-rich tremolite, (Ca_2_(Mg_5.0-4.5_Fe^2+^_0.0-0.5_)Si_8_O2_2_(OH)_2_, and iron-rich ferro-actinolite, Ca_2_(Mg_2.5_-_0.0_Fe^2+^_2.5-5.0_)Si_8_O2_2_(OH)_2_).

Because XRD-analysis are only semi-quantitative, no statistical tests were performed to attempt to explain the observed microbial diversity by using the XRD data as an explanatory variable.

### Major Elements

The composition of the major elements in the sediments (Table 4) generally agrees with the XRD results. Silicate is the dominant element which reflects the dominance of quartz in the sediment samples. The high contents of aluminum and iron are partially explained by their presence in feldspars and amphiboles (respectively). However, since no iron-oxides were detected by XRD it may be possible that much of the iron in the sediments is in an amorphous state. Phosphate was not detected in any of the sediment samples, indicating that its concentration is very low in the whole region.

**Table 4:** Major Elements (weight %) analyzed by ICP-OES for all sediments samples from Lake Cowan (C), Lake Dundas (D), Lake Lefroy (L), and Lake Yellowdine (Y).

	SiO_2_	Al_2_O_3_	Fe_2_O_3_	MnO	MgO	CaO	Na2O	K_2_O	TiO_2_	P_2_O_5_	PF
C1	73.72	7.09	3.84	0.07	3.28	2.39	2.88	0.91	0.66	L.D.	4.82
C2	67.82	8.41	5.06	0.07	4.04	2.23	3.44	0.94	0.64	L.D.	7.61
C3	65.81	8.84	5.29	0.07	4.57	2.11	3.53	1.03	0.59	L.D.	7.80
C4	64.22	8.85	6.00	0.08	4.22	2.53	3.88	0.98	0.75	L.D.	8.58
D1	51.62	9.72	13.10	0.24	5.79	4.13	4.00	0.64	1.30	L.D.	8.33
D2	55.59	7.45	9.86	0.14	3.24	3.11	4.51	1.02	1.58	L.D.	12.00
D3	52.10	11.12	10.86	0.23	3.68	2.30	5.12	1.32	1.46	L.D.	10.92
D4	44.53	6.33	8.51	0.15	2.56	9.54	3.11	0.85	1.43	L.D.	21.58
D5	41.68	7.80	8.50	0.24	2.44	9.81	3.49	0.75	1.32	L.D.	22.98
L1	63.05	7.89	8.64	0.06	3.37	1.16	3.39	0.65	0.56	L.D.	10.24
L2	58.15	8.63	13.37	0.10	3.11	0.77	3.69	0.74	1.02	L.D.	9.55
L3	59.64	7.24	7.91	0.06	3.12	2.50	4.53	0.59	0.58	L.D.	12.68
L4	58.66	8.00	13.81	0.10	2.96	0.79	3.70	0.69	1.07	L.D.	10.56
L5	55.03	9.69	8.76	0.10	4.05	1.87	4.08	0.75	0.70	L.D.	13.62
L6	57.48	8.32	13.82	0.10	3.14	0.75	3.83	0.72	0.97	L.D.	10.00
V1	52.15	15.29	8.24	0.15	5.44	2.56	2.09	1.67	0.55	L.D.	10.51
V2	47.36	18.02	6.73	0.04	2.78	0.95	4.12	1.75	0.55	L.D.	16.43
V3	74.37	6.95	3.27	0.05	3.06	2.18	2.54	1.01	0.24	L.D.	6.68
V4	53.91	15.67	5.73	0.24	3.25	1.21	3.58	1.91	0.51	L.D.	12.71
V5	63.18	10.66	6.00	0.15	5.65	3.21	2.09	1.27	0.45	L.D.	6.44
V6	84.31	5.05	2.02	0.05	1.95	1.52	1.26	1.00	0.13	L.D.	2.31
V7	53.37	10.04	7.92	0.21	9.83	6.09	3.56	0.75	0.52	L.D.	6.97
V8	46.83	16.01	7.13	0.06	4.63	2.12	4.32	1.50	0.53	L.D.	15.58

*PF, Perte au Feu (ignition loss); L.D., below the detection limit.*

Although the carbonates dolomite and calcite were detected in Lake Cowan by XRD, this is not particularly reflected by the composition of the major elements. Values for calcium and magnesium are not significantly higher in the Cowan samples than in other lake sediments. Likely, certain conditions present specifically in Lake Cowan allow for carbonate precipitation while the other lakes lack these conditions. The high values for calcium for the samples D4 and D5 from Lake Dundas correlate with the high values of gypsum (CaSO_4_) determined for these samples by XRD.

Adonis tests showed significant *p*-values (<0.05) for aluminum, iron, sodium, potassium, and titanium when using the unweighted unifrac analysis, suggesting that these elements may affect the species variation observed within the microbial dataset. Using weighted unifrac analysis, aluminum, iron and potassium gave significant *p*-values, showing that these elements also contribute to the relative species abundances observed within the sediments. However, the effect sizes of the Adonis tests were small (<0.2) indicating that these parameters can only explain small percentages of the total variation observed.

### Iron

**FIGURE 9:**
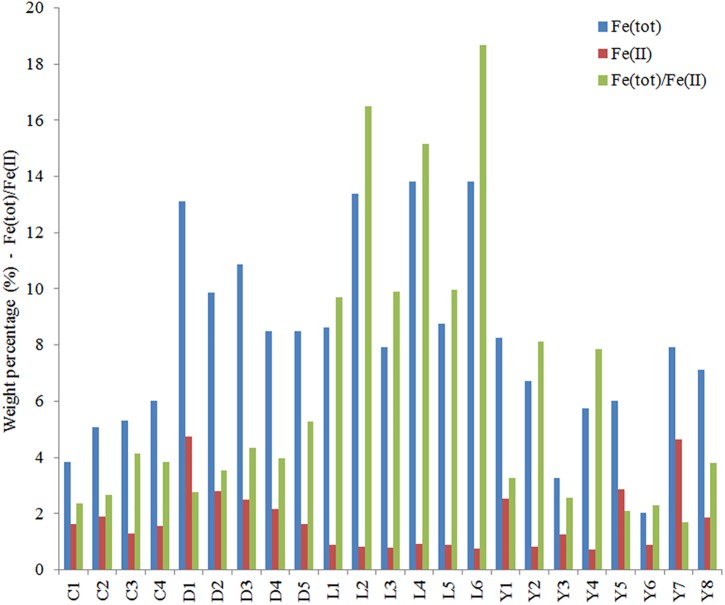
Total iron (blue), ferrous iron (red), and the ratio of total iron [Fe(tot)] and ferrous iron (FeII) determined for samples from the sediments of Lake Cowan, Lake Dundas, Lake Lefroy, and Lake Yellowdine. Values for Fe(tot) and Fe(II) are in weight percentage.

We measured total iron and ferrous iron for all sediment samples, from which the contribution of ferric iron could also be deduced (Figure 9). Total iron is highest in Lake Lefroy and Lake Dundas, contributing up to 14% of the sample weight. Values for these lake sites are often at least two to three times higher than observed in sediments of Lake Yellowdine and Lake Cowan. The ratio between total iron and ferrous iron is also shown in Figure 9. Values for the ratio below 2 indicate a higher contribution of ferrous (Fe^2+^) iron with respect to ferric (Fe^3+^) iron. In all sediments we find higher contributions of oxidized iron than reduced iron. However, this is especially significant in Lake Lefroy and to a lesser degree in Lake Yellowdine where samples, but especially subsurface samples contain much more oxidized iron than reduced iron. Total iron in the sediments had a significant effect on the microbial species that were present in the samples (ADONIS: unweighted unifrac, *R*^2^ = 0.078, *p* = 0.041) as well as on the relative contribution of these species in the (ADONIS: weighted unifrac, *R*^2^ = 0.257, *p* = 0.003).

### Trace Elements

We determined the concentrations of 44 trace elements in each of the sediment samples (Supplementary Figure S3 and Supplementary Table S2). The most abundant trace elements, strontium, chromium and zirconium were present in all lake environments albeit with different abundances. Strontium for example was very abundant in several samples from Lake Dundas while barium and chromium were especially abundant in Lake Cowan and Lake Lefroy. Sediments from Lake Lefroy and Lake Dundas contained relatively high arsenic concentrations when compared to the other sediments (up to 20 times higher). Most of the trace elements did not significantly correlate to variations within the microbial communities. The most notable exception was arsenic (Adonis: unweighted unifrac, *R*^2^ = 0.136, *p* = 0.002; weighted unifrac, *R*^2^ = 0.236, *p* = 0.003).

## Discussion

### Microbial Communities

All four investigated lake environments were found to harbor versatile prokaryotic communities. The biomass we detected, although not exceedingly high, should be easily detectable by a variety of techniques. However, it must be noted that genomic material was generally significantly less abundant in the subsurface. This is especially relevant in the context of planned Mars missions that aim to drill to greater depths in search for organic molecules. Organic carbon (0.04–0.5%) and nitrogen (0.003–0.03%) concentrations were low while phosphorus could not be detected at all. Low organic carbon values were also reported in a previous study investigating two salt lakes in Western Australia (Ruecker et al., 2016) suggesting that this is a regional phenomenon. Low organic carbon in the area likely limits the potential for increased biomass production, especially since our 16S sequence library is dominated by heterotrophic organisms. The same can be assumed for phosphorous which is often a limiting factor in ecosystems (Elser et al., 2007). The C/N ratios vary considerably between samples (between 8 and 22), even when sampled at close proximity. In lacustrine environments, these ratios are influenced by blooming of algae (which are rich in nitrogen) during periods of flooding, while vascular plants (which have lower nitrogen) dominate during episodes of drought (Meyers and Ishiwatari, 1993). The pattern observed here suggests that such flooding and evaporation events can affect the C/N ratios on a very local scale, inducing differences between sediments in close proximity or even at the same location but at different depth.

The prokaryotic communities in the sediments consist of extremely halophilic organisms, as well as slightly halophilic/halotolerant and non-halophilic organisms. This may well be the consequence of the dynamic nature of ephemeral lakes in general, which undergo cycles of flooding and evaporation that cause variations in concentrations of solutes in the waters (Benison and Bowen, 2006). Succession of different microbes likely takes place according to varying levels of solutes, which has also been described for other saline lake environments (Podell et al., 2014) and could explain the diverse phylotypes we observe in the sediments. Isotopic studies on constituents of the lake waters in the Yilgarn craton have shown a marine origin for many of these particles and were likely transported to the inland waters as sea spray aerosols (Hingston and Gailitis, 1976; Bowen and Benison, 2009). This phenomenon also explains the high abundance and variety of prokaryotic phylotypes with a marine origin that we detect in our sequence library.

Prominent detection of halite in the sediments by our XRD analysis indicates salt-rich environments, which is further confirmed by the high abundances of extremely halophilic phylotypes within the sequence dataset. Since the focus of this study was on the characteristics of the sediments, no measurements were done on the lake waters. However, previous studies have reported salinity values for the waters of several of the environments investigated here. It should be noted though that due to strong fluctuations in the volumes of the water bodies these values are not *per se* representative for the specific sample locations and time points in our study. Bowen and Benison (2009) report salinity values (total dissolved solids) for Lake Lefroy (5 g/L), Lake Dundas (100 g/L), and Lake Cowan (26–100 g/L). For Lake Cowan, the salinity was determined on two different locations, which explains the wide range. Salinity values determined by Bowen and Benison (2009) for Lake Lefroy are very low and are not in line with our observations on site nor with our own results. Also, Clarke (1994b) determined total dissolved solids values for surface waters of Lake Lefroy as high as 375 g/L while brines measured 462 g/L, confirming that strong variations can be expected over time and distance within the same lake environment. We could not find any previous literature regarding the salinity values for Lake Yellowdine waters. However, XRD analysis revealed some of the highest detected abundances of halite in the sediments of Lake Yellowdine and during fieldwork, salt deposits were seen throughout the whole lake setting (as was the case for all four lakes).

The most dominant OTUs in the dataset are related to (extremely) halophilic and halotolerant microorganisms, of which members of the *Halobacteriaceae* family are the most prominent. This family comprises 47 known genera with a total of 165 species of which many have a surprising metabolic versatility and unusual characteristics that may assist in their survival or even growth on Mars (Oren, 2014). *Halobacteriaceae* also developed a specialized strategy to cope with high salt levels, known as the ‘salt-in’ strategy (Lanyi, 1974). To counteract the osmotic pressure, cells accumulate high concentrations of K^+^ and Cl^-^ in their interior. The evolutionary development of this strategy aligns with considerable adaptations in the protein and enzyme machinery of these species. Besides *Halobacteriaceae*, only few other known species are known to use this strategy, which include the extremely halophilic bacteria *Salinibacter and* members of the order *Halanaerobiales* (Oren et al., 2002; Oren, 2013b). Notably, Salinibacters are also significantly represented in the sediments that we investigated while *Halanaerobiales* are only detected in low numbers. Although the preferred type of metabolism is heterotrophic with aerobic respiration, members of the *Halobacteriaceae* family can grow in the absence of oxygen by utilizing a specialized light driven proton pump called bacteriorhodopsin or by using alternative electron acceptors such as nitrate or even perchlorate (which is abundantly present in the Martian soil) (Hartmann et al., 1980; Hecht et al., 2009; Oren, 2013a). Certain members of the *Halobacteriaceae* are adapted to survive extreme conditions such as the vacuum of space and large doses of cosmic radiation but are also able to survive for extended periods of time as dormant cells in salt crystals (Oren, 2014). Of special interest for this environment due to the low concentration of phosphorus, is the ability of several *Halobacteriaceae* members to solubilize phosphor by production of organic acids (and thus locally lowering pH) (Yadav et al., 2015). This would allow them to free phosphorous from otherwise insoluble minerals.

While many of the lake waters in the region are acidic and ground-waters also tend to have low pH (Bowen and Benison, 2009), the environments we investigated are more representative for their neutral or slightly acidic counterparts. Previous reports stated pH values between 6 and 7 for Lake Dundas, Lake Lefroy, and Lake Cowan (Lyons et al., 1990; Clarke, 1994b). These values are well in line with the pH values of our sediment samples. No reports were found on the pH of Lake Yellowdine waters but the pH values we determined for the sediments are in the same range as the other samples, suggesting that also this environment has slightly acidic to neutral waters. The more neutral nature of these lake environment is underlined by the composition of the microbial communities. Only 0.4% of the reads belongs to the phylum of *Acidobacteria* while some minor contributions of other potential acidophiles were observed. Even more so, a relatively large fraction of the sequences belongs to phylotypes that thrive under slightly alkaline conditions. These include a *Halobacteriaceae* family member *Natronomonas* (optimum pH 8.5) which makes up 5.5% of the total reads, and an extremely radiation-resistant bacterium (Slade and Radman, 2011) of the genus *Truepera* (optimum pH 7.5–9) from the Phylum *Deinococcus–Thermus*, representing 1.1% of the reads. The optimum pH for the family of *Halobacteriaceae* is also around pH 7 suggesting that the acidity of regional ground waters does not have a direct effect on the microbial communities in the investigated sediments. A previous study by Johnson et al. (2015) focused on the metagenome of the prokaryotic communities in sediment samples of an acidic (pH 3.7) salt lake located about 100 km south of our Lake Dundas sampling site. They found only a small contribution of Archaea to the prokaryotic community but did find that these were dominated by members of the *Halobacteriaceae* family showing that similar phylotypes are also found under very different conditions.

Many of the species we detected are present in the majority of the samples, although at different relative abundances. The largest difficulty in explaining the observed microbial profiles in the samples was that variation within the same lake environment was often found to be equal to (or greater than) variation between different lake environments. This also holds true for many of the environmental parameters that were measured, which complicated correlation of environmental factors to observed microbial variation. Ordination of the samples based on their microbial consortia confirmed the observed patchiness and revealed an image that suggested strong overlap between the different lake environments, while simultaneously strong heterogenic dispersion of the samples was observed.

Part of the observed intra-lake variation in mineralogy and micro-organism diversity may well relate to sedimentary facies. Field observations indicated that the lake environments consist for a large part of mud or silt-flats surrounding topographic lows. There are areas where run-off is concentrated and where groundwater springs enter the lakes. During times of water influx, the salinity of the lakes is likely to be lower with shallow areas of the mud-flats receiving more light. As evaporation occurs, the water recedes toward low-lying areas becoming increasingly saline. The scattered ground-water springs may also provide higher salinity areas of the surrounding mudflats resulting in even more different sub-environments in relative close proximity. Furthermore, fine grained soils (along with microbes within these soils) are scattered by aeolian transport. This would introduce similar microbial communities as well as soil composition patterns to the upper soil layer of the whole region, while simultaneously resulting in unpredictable small-scale variations. The effects of wind on the landscape in this region were previously mentioned by Benison et al. (2007). Detection of elevated quartz in all sediments, even when not located to high Si bedrocks, also suggests that storms, or transportation by water affects the regional composition of (at least) the top layers of the soils.

Some of the environmental parameters such as sampling depth, carbon and nitrogen content, and several of the major elements (e.g., iron, potassium, aluminum) explained the variation in the microbial landscape to some degree. Surface samples often shared more similar species profiles as was also the case for subsurface samples. Elevated levels of iron (as well as arsenic) in lakes Lefroy and Dundas may partially explain why these two environments were more similar to each other in terms of species composition. Aluminum and potassium were significantly higher in Lake Yellowdine sediments which may explain some of the dispersion observed in the ordination. Carbon and nitrogen were also shown to explain a small part of the variation observed within the microbial data but no pattern for this effect was found. Although statistically significant, we were not able to discern the exact nature of these effects or correlate it to the presence or absence of specific phylotypes in individual samples. Due to the dynamic nature of the four lake environments as well as potential interaction between them by groundwater systems and aeolian transport, specific parameters that may influence the communities per sample site were largely obscured.

### Geology

Lakes Lefroy, Cowan, and Dundas are located in the eastern part of the Yilgarn craton between Kalgoorlie and Esperance where two paleodrainage channels interact. Lake Lefroy occurs in the Lefroy paleodrainage channel, while Lakes Cowan and Dundas occur within the Cowan paleodrainage channel (Clarke, 1994a). ‘Yellowdine lake’ is situated about 200 km to the west on the southern border of the Yellowdine nature reserve close to an old gold mine (Figure 1). The bulk mineralogy is generally made up by the same components for each lake environment. Notable exceptions are the selective presence of gypsum (Dundas and Lefroy), clays (Lefroy and Yellowdine), minor carbonates (Cowan) and some variations in the relative abundances of the major components in general. Higher proportions of amphiboles in Lake Dundas and Lake Yellowdine may be connected to the presence of prominent metamorphic rock formations in the direct vicinity of the lakes, which is not present at the other two lake sites. Sediments from Lake Cowan have the highest average crystallinity index and are the only samples that contain carbonates. They also don’t show any signs of the presence of clays and have less amphiboles. The elevated quartz in Lake Cowan does not correlate well with the local gabbroic and basaltic bedrock and suggests inputs from a more distant drainage area. *Marinobacter* species, which were found in high abundances in two sediment samples from Lake Cowan (C3 and C4) have previously been shown to be involved in carbonate precipitation (Sánchez-Román et al., 2007). This may explain the detection of calcite and dolomite in Lake Cowan. However, *Marinobacter species* are also detected in other samples (although in somewhat lower quantities) while no carbonates are detected there. No significant effect of carbonates on the microbial community structure was found, making it more likely that the presence of carbonates results from a non-biogenic source.

Most of the observed minerals in the sediments are likely detrital while halite, gypsum and possibly the clays could have an authigenic origin. Lake Cowan and Lake Dundas are in close proximity to each other and no clays were detected in any of the sediment samples from these lakes. Kaolinite was detected in Lake Lefroy but was most prominent, together with the other clay illite in Lake Yellowdine, which is located much more to the west than the other three lakes. If the presence of clays would be the sole result of weathering and subsequent aeolian transport, one would expect to detect some clays in the other lake sediments as well. The lack of this would suggest that at least part of the clays detected on the other sites have an authigenic origin.

Although relative high concentrations of iron were measured in the major element analysis, no iron oxides or iron salts were detected by XRD. Absence of iron oxides/salts in the XRD data suggests that they contribute little to the total crystalline mineral assemblage. Therefore, we assume that much of the iron in the soil is present in a mostly amorphous state (and of course as component of the amphiboles). However, we found that a large fraction of the iron is in its oxidized state which would generally cause it to precipitate as minerals such as ferrihydrite. Ferrihydrite however, is often X-ray amorphous prior to transformation to goethite, which could explain why we didn’t detect it during XRD analysis (Houben and Kaufhold, 2018). It is possible that due to the dynamic nature of the lakes, there is simply not enough time for iron hydroxides/oxides to fully transform into goethite or hematite. Johnson et al. (2015) also determined the mineralogical composition of a sediment sample from an acidic (pH 3.7) lake environment and found that only 0.2% of the bulk was composed of iron oxides. Such low abundances would likely not have been detected by our implemented techniques.

All subsurface samples from Lake Lefroy and several from Lake Yellowdine had significantly higher contributions of oxidized iron than the surface samples. Intuitively the opposite would be expected with higher oxidation rates at the surface. It may be possible that due to inflow of regional acidic groundwater (being closer to the subsurface than to the surface), iron is dissolved and subsequently transported away by the underlying drainage systems. This would result in relative higher amounts of ferric iron being left behind in the sediments. Another way to achieve this phenomenon would be by the work of anaerobic iron oxidizing bacteria, which can couple the oxidation of Fe(II) to the reduction of oxidized compounds such as sulfates, nitrates, or even perchlorates (Weber et al., 2006). Although OTUs related to such microorganisms are not clearly identified in the dataset, some 16S sequences are novel to these lake environments and thus perhaps from species with specialized metabolic properties.

Gypsum was mainly present in the sediments of Lake Dundas and Lake Lefroy, although the distribution was very patchy. Its contributions were generally low but were very high in two samples from Lake Dundas (D4 and D5). Notably, these two samples had a higher pH and element analysis revealed high abundances of calcium. Both elevated pH and elevated concentration of calcium would facilitate precipitation of gypsum from the lake waters. The variation in evaporite mineralogy occurs owing to the solubility of minerals in the fluids. The solubility increases in the order from carbonate to gypsum to halite. Precipitation of halite tends to occur after the most evaporation, although it is dependent on the initial composition of the water. The conditions resulting in the formation of gypsum (but also carbonates and halite) thus strongly relate to the degree of evaporation, addition of freshwater by run-off and precipitation. The detection of gypsum may indicate that the remainder of the water body has increased concentrations of its constituents (i.e., halite), in turn potentially affecting the composition of microbial communities on site. Indeed, we do observe consistently higher contributions of the extremely halophilic *Halobacteriaceae* family members in all samples from Lake Dundas and Lake Lefroy, which could indicate that at the time that the water retracted from this area, it had a higher salinity.

Furthermore, comparing the major element analysis to the XRD results shows that the two datasets are generally in agreement. Silicates, iron and aluminum are the dominant elements reflecting the dominant mineral phases. Magnesium, calcium, and sodium also contribute significantly to the major elements as would be expected based on the mineralogy of the samples. Trace element analysis showed the presence of a large variety of elements with some in very high concentrations (i.e., strontium, chromium, barium, nickel, zirconium). Although we saw some variations between the different lakes in the concentrations of certain trace elements, these are relatively minor. This is likely because the regional ground-waters dissolve and transport such elements throughout the region where they are locally deposited in the sediments (Mann, 1983).

### Prospects for Mars Related Research

Although the main focus for Mars-related research in this region has been on acidic lakes, we found that these slightly acidic, to neutral lake environments also have potential as analogs for certain Martian sites. The amount and diversity of life we found in these four Australian lake environments has implications for the search for life in our solar system. Microorganisms with flexibility in their metabolism (such as *Halobacteriaceae*) can be considered as viable candidates when looking for life, or remnants of ancient life on Mars (Oren, 2014). Due to their extremely halophilic nature they may theoretically be able to survive in liquid brines that are (and once were) present on Mars. Although cosmic radiation will destroy organic molecules near the surface of Mars, a recent study suggests the presence of a large subsurface lake underneath an ice cap in the Planum Australe (Orosei et al., 2018). The existence of liquid water at this location is suggested to arise owing to high salinity. Halophilic organisms, such as those observed in this study, are uniquely adapted to such conditions and thus potentially provide analogies to putative microorganisms in such environments, although the low temperatures of the water would require cold-adapted strategies.

All of the minerals reported in this study have been previously reported to occur on Mars (Ehlmann et al., 2011; Carter et al., 2013). Putative salt flats have been identified at several locations, where they left behind sulfate and chloride-rich deposits (Osterloo et al., 2010; Flahaut et al., 2015). It is challenging to determine the exact composition of the sediments left in those settings due to the limited spatial resolution of remote sensing instruments, and the presence of obscuring crusts and dust. However, it is important to note that Mars crust is rather basaltic (McSween et al., 2003), and resulting sediments might contain less Si, Al, and Ca, but more Fe and Mg than the Australian examples studied here.

Several of the more acidic lakes in the region were previously described as good analogs for the Mars Burns formation (Benison and Bowen, 2006). This comparison was in part based on prominent detection of minerals such as jarosite, goethite, and hematite, which were not detected in our study. However, ferrihydrite is a precursor for goethite and hematite, and given the right conditions and timescale it is possible that these minerals will be formed in the authigenic process. Also, we focused on lake environments with slightly acidic to neutral pH while many of the features in the Mars Burns formation are proposed to have formed in acidic waters (Benison and Bowen, 2006). Environments on Mars however, are not uniformly acidic and other regions such as Yellowknife bay in the Gale crater have likely formed in near neutral pH waters (Grotzinger et al., 2014), and may be more closely related to the lake settings we investigated here. In 2017, new data regarding the long-term presence of an ancient lake in Gale crater (between 3.1 and 3.8 billion years ago) revealed that conditions were favorable to support a wide range of different microbial metabolisms simultaneously (Hurowitz et al., 2017), something which we also observe in the investigated lake settings here.

In the Yilgarn Craton, significant differences in pH and salinity exist between lakes that are in close proximity (Benison et al., 2007), which will generally result in spatial variations in the deposition of minerals. Furthermore, variations we found within individual lakes also indicate localized sub-environments related to sedimentary facies. This, for example would be the case with the most hypohaline conditions experienced by depocenters in which the most saline solutions are concentrated by evaporation, whilst more neutral water prevails on the surrounding mud and silt flats. Over time, these intra- and inter-lake variations will likely cause a highly variable deposition of minerals and sedimentary features in the geological record. A similar observation was found in the stratified rock record of the Gale crater, although differences in deposition were explained by aerobic versus anaerobic conditions. Furthermore, the same study found that the investigated facies were overlain by a saline overprint after burial and lithification (Hurowitz et al., 2017). Halite is the dominant evaporite we found on the four lake sites and is likely to induce similar features in the rock record after long-term desiccation events.

Furthermore, halite (as well as gypsum) is an excellent biomarker preserver and has been shown to be widely present on Mars (Osterloo et al., 2010), making halite crusts a priority target for future life detection missions on the red planet. High salinity levels in water (preferably in combination with higher pressures) also lowers the freezing point of water substantially (to as low as -50°C in saturated brines) (Brass, 1980; Lide, 2004). Due to the freezing temperatures on Mars, salts are of vital importance to allow for the presence of liquid water. Of equal importance is that saline water has a lower triple point according to Raoult’s law, making it more resistant to sublimation and thus less likely to directly dissipate into Mars’ atmosphere in gas form. Although the lake sites investigated in this study are prone to varying ranges of salinity due to their ephemeral nature, one can assume that salinity levels are highest during desiccation events. Depending in part on the extent of evaporation, extremely concentrated brines can form in these environments and according to our sequence results there are many species present that are able to survive in such brines. If eventually water would evaporate completely, salt crystals are formed which could trap and preserve remaining microorganisms and biomolecules inside. Targeting salt flats and other salt rich environments on Mars should be considered for future life detection missions.

However, the dynamic interaction between groundwater systems and surface environments as seen in the Yilgarn craton is something that likely did not happen on ancient Mars. Observed drainage systems and river beddings on Mars are more likely to be results of episodic outburst floods rather than a continuous supply of groundwater to a surface region (Carr, 1979). This would logically result in more short-lived aqueous environments with a less steady supply of nutrients and trace elements for potentially present microorganisms.

## Conclusion

Our study differed from most previous studies done in the region since we focused on lake environments with slightly acidic to neutral pH. Therefore, these environments serve mostly as analogs to Mars in a phase where acidification of the surface waters had not yet fully set in. The detailed analysis of the prokaryotic communities we provide contributes to the growing knowledge on the type of life that can be found in such environments, which we found to be dominated by extremely halophilic organisms. Organic carbon and nitrogen load was generally very low but varied strongly between different samples, as did the C/N ratios. This indicates a patchy distribution of the organic content within these environments and likely affects the distribution of life to a certain degree. The mineralogy we found is typical for an environment that is prone to successive cycles of flooding and evaporation events, with evaporites like halite, gypsum and carbonates detected in a variety of samples. Trace element analysis revealed that most elements were similarly distributed which could be an effect of the complex ground-water system in the region, supplying the different lakes with water rich in metals and other chemical elements. Major element analysis showed high abundance of iron in most of the samples. However, no iron oxides or sulfates were detected by XRD, suggesting that full maturation to minerals such as goethite, hematite or jarosite did not take place. The dynamic nature of these lakes appears to influence the biological, biochemical and geological components of the ecosystem. Inter- and intra-lake variations in the distribution of life were significant, and could only to a minor degree be explained by underlying environmental conditions. Microbial communities may be significantly influenced by small scale local effects caused by variations in geological settings and dynamic interactions caused by aeolian transport and flooding and evaporation events.

## Data Availability

This Targeted Locus Study project has been deposited at DDBJ/EMBL/GenBank under the accession KCZV00000000. The version described in this paper is the first version, KCZV01000000.

## Author Contributions

JA wrote and prepared the manuscript and largely conducted the experimental steps and data analysis. JF assisted with the mineralogical and elemental analysis and subsequent interpretation of the obtained data. ZM, PB, and MG were in charge of the sample collection on site and the initial start of the project as a whole. All co-authors provided valuable feedback and comments regarding data analysis and manuscript preparation.

## Conflict of Interest Statement

The authors declare that the research was conducted in the absence of any commercial or financial relationships that could be construed as a potential conflict of interest.
