# Supplementary material for: Microbial Communities in Sediments From Four Mildly Acidic Ephemeral Salt Lakes in the Yilgarn Craton (Australia) – Terrestrial Analogs to Ancient Mars
Source: Front Microbiol. 2019 May 6;10:779. doi: 10.3389/fmicb.2019.00779 (PMC6512757; doi:10.3389/fmicb.2019.00779)
Supplement: Supplementary file 1 [file Table_1.DOCX]

**Supplementary material**


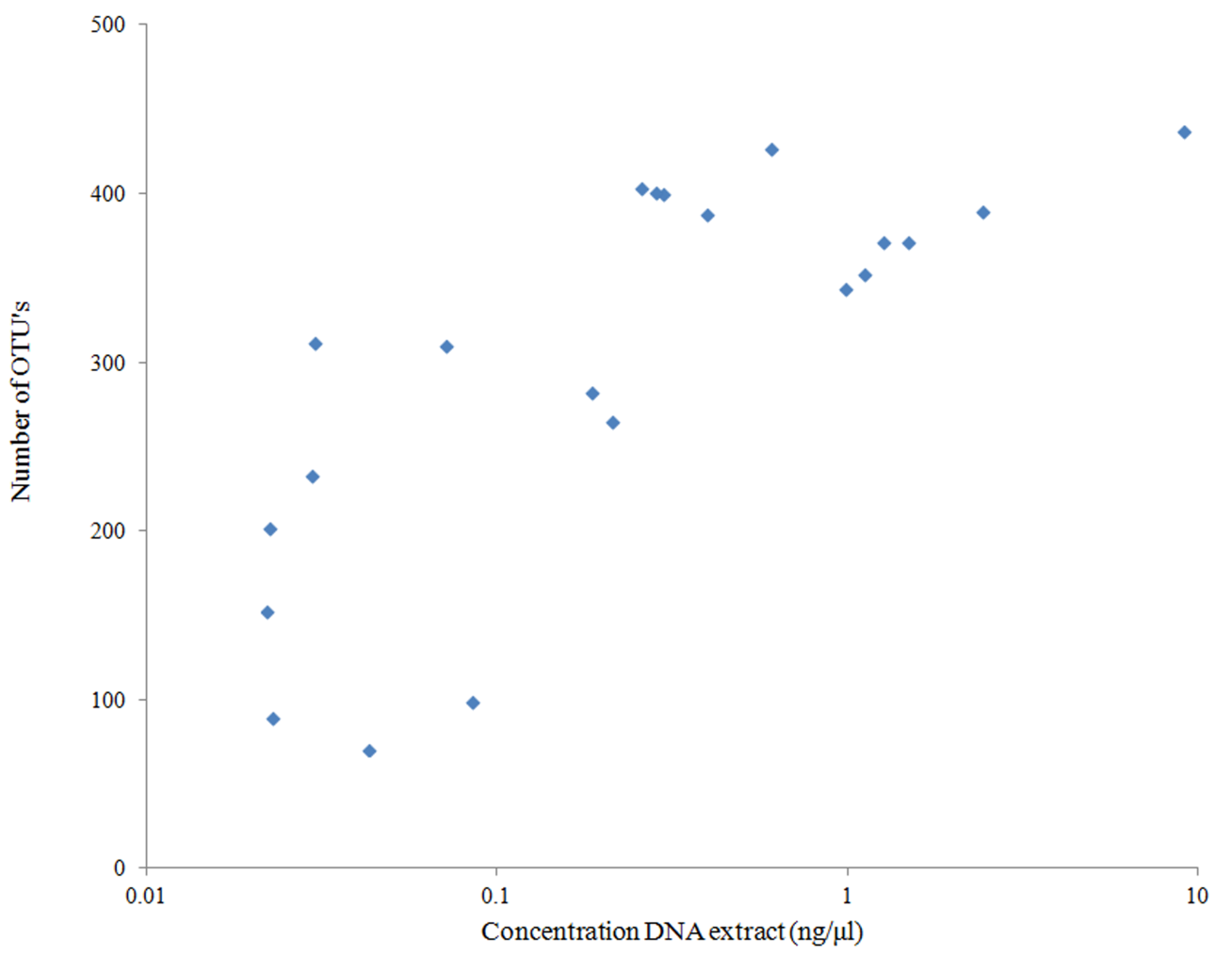


*Figure S1: Correlation between the concentration of DNA in the original extracts and the number of OTUs observed in the rarefied sequence data (alpha diversity)*


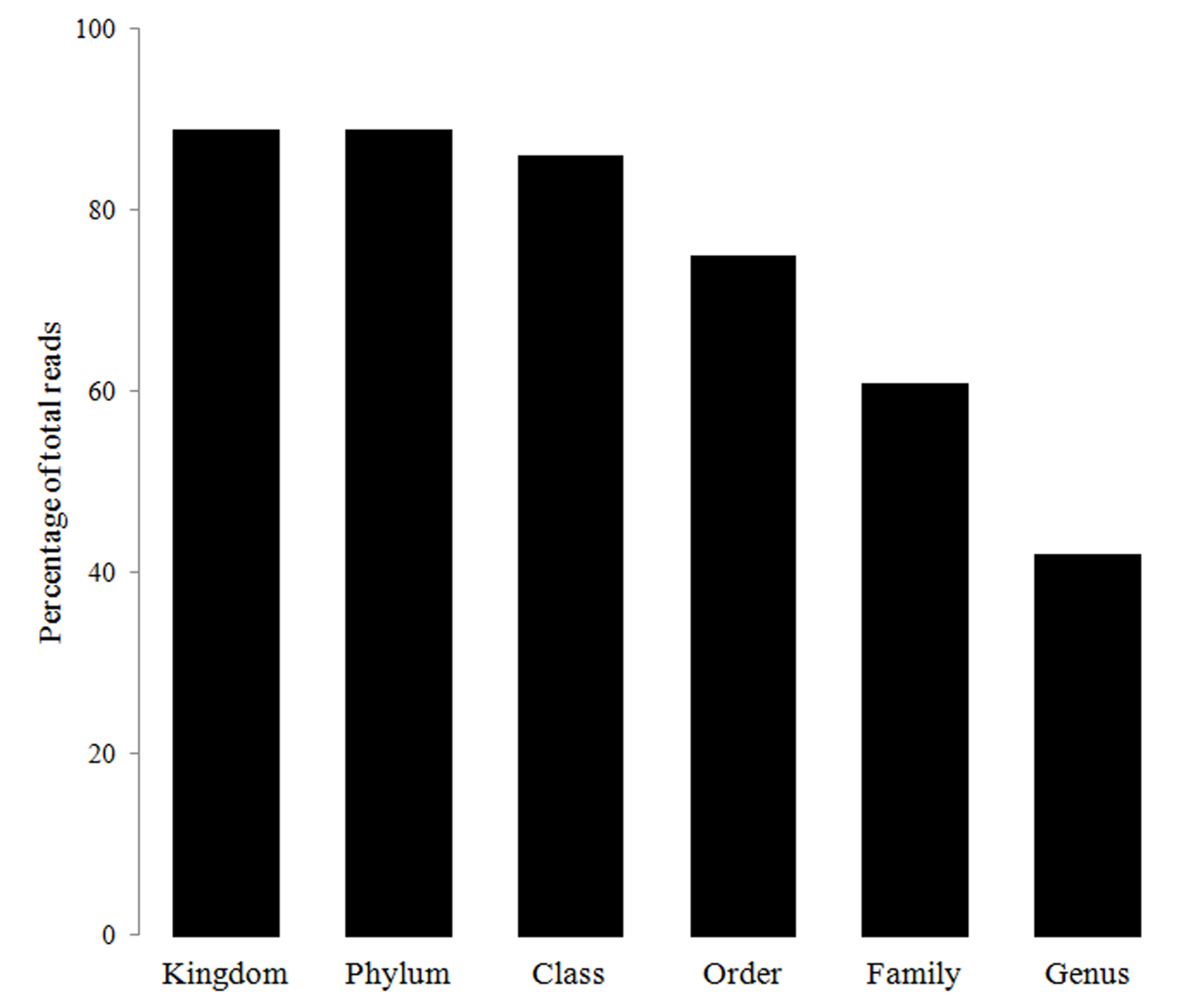


*Figure S2: Percentage of reads assigned to taxonomic rank at 97% similarity*


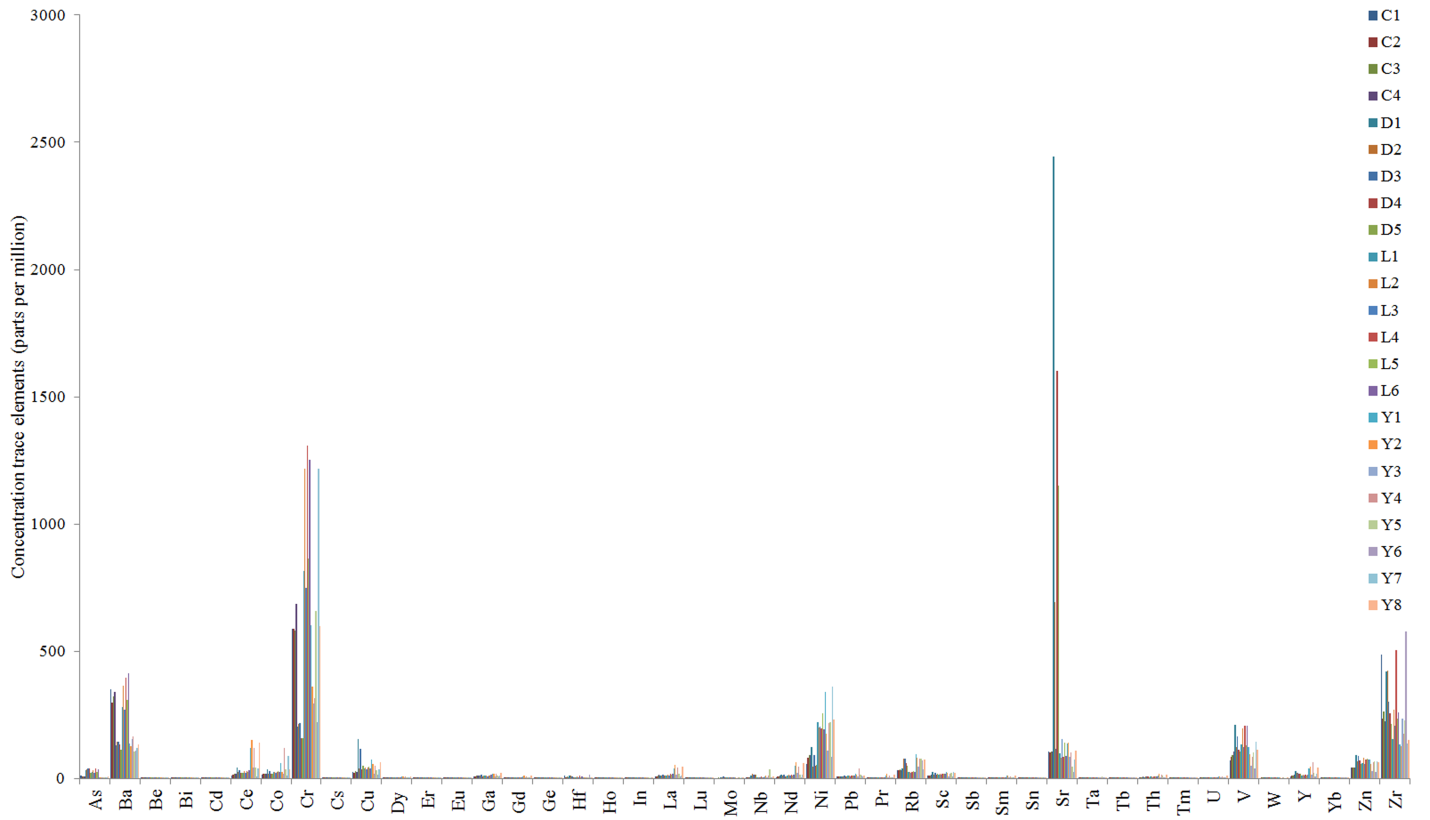


*Figure S3: Trace elements detected by Mass spectroscopy. Values showed in parts per million (ppm). For a detailed overview see table x in the supplementary material. C = Lake Cowan, D = Lake Dundas, L = Lake Lefroy, Y = Lake Yellowdine*

*Table S1: Overview of characteristics of the raw, filtered and rarefied 16S amplicon Illumina dataset*

|  | **Pre-filtering** |  | **Post-filtering** |  | **Rarefied** |
| --- | --- | --- | --- | --- | --- |
| Reads total | 1.817.287 |  | 1.748.123 |  | 51.700 |
| OTUs | 4270 |  | 1315 |  | 1301 |
| Table density | 0.232 |  | 0.448 |  | 0.226 |
| Min | 881 |  | 828 |  | 2350 |
| Max | 143484 |  | 140318 |  | 2350 |
| Median | 94402.5 |  | 93193.5 |  | 2350 |
| Mean | 75720.292 |  | 72838.458 |  | 2350 |
|  |  |  |  |  |  |

*Table S2: Trace elements detected by Mass spectroscopy. Values showed in parts per million (ppm). C = Lake Cowan, D = Lake Dundas, L = Lake Lefroy, Y = Lake Yellowdine.*

|  | **As** | **Ba** | **Be** | **Bi** | **Cd** | **Ce** | **Co** | **Cr** | **Cs** | **Cu** | **Dy** | **Er** | **Eu** | **Ga** | **Gd** | **Ge** | **Hf** |
| --- | --- | --- | --- | --- | --- | --- | --- | --- | --- | --- | --- | --- | --- | --- | --- | --- | --- |
| **C1** | 11.08 | 351.39 | 0.50 | 0.18 | 0.65 | 10.68 | 13.71 | 588.70 | 0.56 | 25.25 | 1.49 | 1.00 | 0.41 | 7.97 | 1.16 | 0.77 | 11.77 |
| **C2** | 8.05 | 298.28 | 0.69 | 0.15 | 0.05 | 16.43 | 17.81 | 589.73 | 0.77 | 21.72 | 1.78 | 1.13 | 0.49 | 9.23 | 1.57 | 0.87 | 5.83 |
| **C3** | 7.62 | 324.15 | 0.65 | 0.15 | 0.04 | 17.92 | 19.06 | 580.99 | 0.77 | 27.39 | 1.97 | 1.22 | 0.56 | 11.23 | 1.79 | 0.88 | 6.54 |
| **C4** | 8.12 | 338.89 | 0.54 | 0.19 | 0.05 | 20.17 | 19.71 | 685.41 | 0.74 | 25.56 | 2.43 | 1.52 | 0.66 | 10.96 | 2.19 | 0.97 | 5.91 |
| **D1** | 33.37 | 130.19 | 0.65 | 0.48 | 0.14 | 43.18 | 35.14 | 204.88 | 1.37 | 154.88 | 4.61 | 2.91 | 0.97 | 13.06 | 4.11 | 1.18 | 10.09 |
| **D2** | 36.47 | 144.16 | 0.71 | 0.85 | 0.09 | 26.77 | 19.39 | 215.85 | 1.79 | 39.13 | 3.73 | 2.25 | 0.64 | 10.48 | 3.11 | 1.21 | 10.45 |
| **D3** | 39.31 | 145.67 | 1.00 | 0.84 | 0.10 | 33.34 | 29.11 | 217.84 | 2.23 | 117.50 | 4.18 | 2.58 | 0.93 | 15.30 | 3.64 | 1.35 | 7.64 |
| **D4** | 38.35 | 132.76 | 0.77 | 0.79 | 0.09 | 21.18 | 19.63 | 159.97 | 1.65 | 35.80 | 2.96 | 1.89 | 0.57 | 9.54 | 2.50 | 0.90 | 6.30 |
| **D5** | 22.13 | 111.33 | 1.48 | 0.57 | 0.08 | 20.67 | 18.58 | 157.91 | 1.43 | 50.05 | 2.74 | 1.73 | 0.54 | 10.48 | 2.32 | 0.78 | 5.39 |
| **L1** | 25.59 | 280.53 | 0.52 | 0.21 | 0.06 | 23.65 | 24.58 | 816.03 | 1.29 | 37.97 | 2.12 | 1.24 | 0.64 | 10.99 | 2.53 | 0.99 | 4.01 |
| **L2** | 32.76 | 365.18 | 0.73 | 0.30 | 0.07 | 29.49 | 25.21 | 1216.29 | 1.24 | 42.09 | 2.81 | 1.68 | 0.80 | 12.74 | 2.66 | 1.06 | 6.62 |
| **L3** | 23.54 | 269.14 | 0.47 | 0.21 | 0.06 | 20.92 | 22.40 | 749.34 | 1.05 | 34.89 | 1.92 | 1.16 | 0.55 | 9.67 | 1.83 | 0.90 | 5.33 |
| **L4** | 37.99 | 396.29 | 0.62 | 0.33 | 0.14 | 30.39 | 24.23 | 1307.65 | 1.13 | 44.57 | 2.92 | 1.70 | 0.82 | 12.50 | 2.70 | 1.10 | 12.12 |
| **L5** | 26.32 | 308.83 | 0.65 | 0.24 | 0.06 | 27.15 | 29.87 | 865.31 | 1.32 | 46.80 | 2.37 | 1.39 | 0.70 | 12.91 | 2.28 | 1.08 | 5.88 |
| **L6** | 36.10 | 411.80 | 0.62 | 0.33 | 0.08 | 31.46 | 24.29 | 1251.66 | 1.19 | 40.87 | 2.78 | 1.63 | 0.82 | 13.74 | 2.74 | 1.10 | 6.53 |
| **Y1** | 6.03 | 135.92 | 3.13 | 0.53 | 0.07 | 118.66 | 61.03 | 603.81 | 4.50 | 73.37 | 7.51 | 3.92 | 2.17 | 17.24 | 8.43 | 1.58 | 3.71 |
| **Y2** | 5.27 | 126.74 | 3.35 | 0.75 | 0.05 | 151.21 | 26.98 | 361.18 | 3.56 | 58.00 | 8.99 | 4.30 | 2.61 | 20.30 | 10.48 | 1.66 | 3.65 |
| **Y3** | 3.11 | 156.58 | 1.32 | 0.18 | 0.05 | 42.41 | 19.43 | 294.18 | 1.19 | 19.18 | 3.08 | 1.61 | 0.95 | 7.53 | 3.59 | 0.97 | 5.82 |
| **Y4** | 4.84 | 163.99 | 2.89 | 0.58 | 0.05 | 118.92 | 121.16 | 314.26 | 3.09 | 49.43 | 9.22 | 5.28 | 2.17 | 18.15 | 9.39 | 1.52 | 4.92 |
| **Y5** | 3.52 | 106.96 | 1.31 | 0.37 | 0.07 | 41.66 | 34.41 | 657.09 | 2.35 | 34.15 | 3.35 | 2.08 | 0.63 | 13.02 | 3.05 | 1.19 | 5.91 |
| **Y6** | 2.45 | 107.88 | 0.52 | 0.17 | 1.60 | 9.62 | 10.51 | 222.69 | 1.80 | 16.36 | 0.95 | 0.68 | 0.21 | 6.33 | 0.98 | 0.79 | 13.61 |
| **Y7** | 2.61 | 118.91 | 1.33 | 0.28 | 0.09 | 40.93 | 88.16 | 1216.58 | 1.20 | 36.47 | 3.15 | 1.93 | 0.74 | 11.69 | 2.77 | 1.22 | 3.54 |
| **Y8** | 7.15 | 134.50 | 3.36 | 0.86 | 0.06 | 139.42 | 35.19 | 598.64 | 2.98 | 63.52 | 8.55 | 4.07 | 2.59 | 20.73 | 10.02 | 1.62 | 4.13 |

*Table S2 (continued): Trace elements detected by Mass spectroscopy. Values showed in parts per million (ppm). C = Lake Cowan, D = Lake Dundas, L = Lake Lefroy, Y = Lake Yellowdine.*

|  | **Ho** | **In** | **La** | **Lu** | **Mo** | **Nb** | **Nd** | **Ni** | **Pb** | **Pr** | **Rb** | **Sc** | **Sb** | **Sm** | **Sn** |
| --- | --- | --- | --- | --- | --- | --- | --- | --- | --- | --- | --- | --- | --- | --- | --- |
| **C1** | 0.34 | 0.03 | 6.51 | 0.19 | .D. | 4.14 | 4.90 | 57.93 | 8.70 | 1.29 | 31.21 | 10.18 | 0.45 | 1.14 | 0.91 |
| **C2** | 0.39 | 0.04 | 9.13 | 0.18 | L.D. | 4.42 | 7.51 | 81.39 | 8.34 | 1.98 | 32.59 | 13.09 | 0.45 | 1.64 | 1.22 |
| **C3** | 0.43 | 0.04 | 11.95 | 0.20 | 0.62 | 4.39 | 8.90 | 93.42 | 9.51 | 2.31 | 35.95 | 13.24 | 0.45 | 1.93 | 1.13 |
| **C4** | 0.55 | 0.05 | 14.62 | 0.24 | L.D. | 5.27 | 10.95 | 90.47 | 8.32 | 2.88 | 34.03 | 14.77 | 0.49 | 2.29 | 1.28 |
| **D1** | 1.02 | 0.06 | 11.44 | 0.49 | 3.23 | 12.48 | 14.41 | 124.71 | 9.58 | 3.33 | 41.11 | 24.35 | 0.63 | 3.79 | 1.67 |
| **D2** | 0.80 | 0.05 | 13.06 | 0.38 | 1.38 | 16.84 | 12.78 | 47.35 | 9.45 | 3.26 | 78.44 | 14.88 | 0.49 | 3.13 | 4.23 |
| **D3** | 0.91 | 0.07 | 16.47 | 0.42 | 7.61 | 14.52 | 15.69 | 90.86 | 11.68 | 4.01 | 79.47 | 23.24 | 0.69 | 3.71 | 2.92 |
| **D4** | 0.65 | 0.05 | 10.84 | 0.32 | 2.68 | 14.07 | 9.75 | 50.16 | 9.26 | 2.51 | 60.38 | 13.55 | 0.43 | 2.44 | 1.76 |
| **D5** | 0.61 | 0.05 | 10.42 | 0.28 | 4.41 | 15.57 | 9.35 | 53.36 | 8.65 | 2.38 | 50.26 | 18.01 | 0.34 | 2.24 | 1.22 |
| **L1** | 0.45 | 0.05 | 12.84 | 0.48 | 0.69 | 3.75 | 11.28 | 221.59 | 11.45 | 2.97 | 24.89 | 14.95 | 1.08 | 2.30 | 1.45 |
| **L2** | 0.60 | 0.07 | 16.67 | 0.27 | 0.94 | 5.58 | 14.74 | 199.45 | 12.51 | 3.93 | 25.73 | 18.17 | 1.68 | 3.11 | 1.81 |
| **L3** | 0.42 | 0.05 | 11.26 | 0.20 | 0.86 | 3.50 | 9.95 | 203.40 | 9.45 | 2.56 | 20.66 | 13.50 | 1.00 | 2.02 | 1.15 |
| **L4** | 0.60 | 0.07 | 17.59 | 0.29 | 1.07 | 6.39 | 15.32 | 197.49 | 13.04 | 4.08 | 24.11 | 17.36 | 1.68 | 3.09 | 1.73 |
| **L5** | 0.50 | 0.07 | 14.54 | 0.23 | 0.86 | 5.03 | 12.42 | 255.52 | 10.56 | 3.23 | 27.69 | 17.98 | 1.09 | 2.56 | 1.64 |
| **L6** | 0.58 | 0.07 | 19.41 | 0.26 | 1.05 | 5.40 | 15.83 | 192.71 | 13.12 | 4.27 | 24.98 | 17.84 | 1.70 | 3.22 | 1.80 |
| **Y1** | 1.52 | 0.09 | 38.97 | 0.51 | 0.73 | 6.62 | 50.88 | 339.80 | 16.82 | 13.18 | 96.38 | 24.80 | 0.33 | 9.84 | 2.39 |
| **Y2** | 1.75 | 0.12 | 53.97 | 0.52 | 0.89 | 10.49 | 64.97 | 174.26 | 15.08 | 17.17 | 80.65 | 22.21 | 0.34 | 12.28 | 2.93 |
| **Y3** | 0.62 | 0.04 | 14.88 | 0.22 | L.D. | 4.21 | 22.47 | 108.50 | 8.31 | 5.56 | 46.75 | 9.61 | 0.10 | 4.18 | 0.77 |
| **Y4** | 2.00 | 0.09 | 41.39 | 0.68 | 0.80 | 11.78 | 47.85 | 216.16 | 37.79 | 12.46 | 79.50 | 18.96 | 0.26 | 9.37 | 2.41 |
| **Y5** | 0.74 | 0.05 | 19.62 | 0.35 | 0.55 | 36.63 | 16.17 | 222.61 | 13.43 | 4.44 | 78.02 | 20.79 | 0.15 | 3.30 | 1.91 |
| **Y6** | 0.22 | L.D. | 6.52 | 0.17 | L.D. | 1.39 | 4.29 | 85.83 | 12.20 | 1.16 | 70.85 | 6.38 | 0.10 | 0.86 | 1.24 |
| **Y7** | 0.68 | 0.06 | 12.73 | 0.30 | 0.55 | 3.31 | 13.78 | 360.60 | 8.88 | 3.59 | 37.47 | 27.11 | 0.17 | 2.97 | 1.58 |
| **Y8** | 1.63 | 0.13 | 48.25 | 0.50 | 1.58 | 6.61 | 61.71 | 230.29 | 13.17 | 16.27 | 74.12 | 23.04 | 0.34 | 11.73 | 2.84 |

*Table S2 (continued): Trace elements detected by Mass spectroscopy. Values showed in parts per million (ppm). C = Lake Cowan, D = Lake Dundas, L = Lake Lefroy, Y = Lake Yellowdine.*

|  | **Sr** | **Ta** | **Tb** | **Th** | **Tm** | **U** | **V** | **W** | **Y** | **Yb** | **Zn** | **Zr** |
| --- | --- | --- | --- | --- | --- | --- | --- | --- | --- | --- | --- | --- |
| **C1** | 106.81 | 0.61 | 0.21 | 4.74 | 0.16 | 1.03 | 69.89 | < L.D. | 9.16 | 1.19 | 43.88 | 486.28 |
| **C2** | 101.65 | 0.57 | 0.27 | 5.66 | 0.17 | 1.27 | 86.55 | 0.88 | 10.16 | 1.21 | 43.03 | 234.66 |
| **C3** | 106.69 | 0.93 | 0.30 | 6.23 | 0.18 | 1.99 | 91.63 | 0.81 | 11.25 | 1.30 | 43.99 | 261.90 |
| **C4** | 107.34 | 0.85 | 0.37 | 7.79 | 0.22 | 1.64 | 105.27 | 0.99 | 14.56 | 1.59 | 43.87 | 223.34 |
| **D1** | 2442.14 | 2.19 | 0.71 | 4.14 | 0.43 | 1.80 | 209.92 | 5.22 | 30.06 | 3.01 | 90.67 | 418.80 |
| **D2** | 694.12 | 5.86 | 0.57 | 8.25 | 0.35 | 1.99 | 123.12 | 2.25 | 22.21 | 2.50 | 69.13 | 424.20 |
| **D3** | 116.73 | 3.48 | 0.64 | 8.19 | 0.38 | 2.72 | 165.24 | 2.77 | 23.44 | 2.70 | 86.82 | 300.85 |
| **D4** | 1600.03 | 4.54 | 0.44 | 6.75 | 0.28 | 1.67 | 112.08 | 1.95 | 17.72 | 2.04 | 69.45 | 255.66 |
| **D5** | 1151.77 | 6.11 | 0.41 | 5.94 | 0.26 | 1.52 | 106.66 | 2.05 | 17.01 | 1.84 | 57.39 | 213.16 |
| **L1** | 99.68 | 0.40 | 0.32 | 6.34 | 0.18 | 1.03 | 134.21 | 2.10 | 10.99 | 1.27 | 60.11 | 156.09 |
| **L2** | 82.78 | 1.46 | 0.44 | 9.06 | 0.25 | 1.47 | 198.31 | 2.44 | 14.66 | 1.75 | 82.78 | 269.44 |
| **L3** | 155.42 | 0.41 | 0.30 | 5.65 | 0.18 | 0.95 | 121.92 | 1.87 | 10.48 | 1.25 | 56.68 | 208.43 |
| **L4** | 86.38 | 0.95 | 0.44 | 9.21 | 0.27 | 1.57 | 207.84 | 2.96 | 15.05 | 1.80 | 74.28 | 504.88 |
| **L5** | 140.19 | 0.68 | 0.37 | 7.22 | 0.21 | 1.18 | 131.69 | 2.18 | 12.65 | 1.44 | 77.66 | 234.64 |
| **L6** | 88.96 | 0.84 | 0.44 | 9.33 | 0.24 | 1.69 | 206.23 | 2.61 | 14.46 | 1.67 | 75.56 | 258.90 |
| **Y1** | 137.86 | 0.94 | 1.28 | 11.35 | 0.53 | 9.61 | 123.52 | 1.39 | 40.24 | 3.40 | 73.20 | 132.43 |
| **Y2** | 139.90 | 1.21 | 1.56 | 19.94 | 0.57 | 9.76 | 96.44 | 1.78 | 47.85 | 3.66 | 56.45 | 126.11 |
| **Y3** | 83.52 | 0.70 | 0.52 | 4.35 | 0.22 | 3.43 | 50.26 | < L.D. | 16.12 | 1.47 | 27.65 | 236.47 |
| **Y4** | 102.19 | 1.22 | 1.47 | 15.59 | 0.72 | 9.29 | 84.69 | 1.73 | 63.57 | 4.43 | 63.44 | 175.35 |
| **Y5** | 46.89 | 6.52 | 0.51 | 10.90 | 0.32 | 3.07 | 101.33 | 1.30 | 21.52 | 2.26 | 67.02 | 229.60 |
| **Y6** | 24.86 | 0.34 | 0.15 | 3.70 | 0.11 | 1.21 | 39.63 | < L.D. | 8.07 | 0.80 | 26.53 | 579.15 |
| **Y7** | 75.89 | 0.71 | 0.47 | 5.22 | 0.28 | 3.63 | 145.28 | < L.D. | 17.04 | 1.96 | 67.47 | 137.04 |
| **Y8** | 107.77 | 1.49 | 1.50 | 13.36 | 0.54 | 9.95 | 113.39 | 1.85 | 42.81 | 3.48 | 62.97 | 152.23 |
